# α-TubK40me3 is required for neuronal polarization and migration by promoting microtubule formation

**DOI:** 10.1038/s41467-021-24376-2

**Published:** 2021-07-05

**Authors:** Xuan Xie, Shaogang Wang, Mingyi Li, Lei Diao, Xingyu Pan, Jijun Chen, Weiguo Zou, Xu Zhang, Wenfeng Feng, Lan Bao

**Affiliations:** 1 State Key Laboratory of Cell Biology, Shanghai Institute of Biochemistry and Cell Biology, CAS Center for Excellence in Molecular Cell Science, Chinese Academy of Sciences, Shanghai, China; 2 University of Chinese Academy of Sciences, Beijing, China; 3 School of Life Science and Technology, ShanghaiTech University, Shanghai, China; 4 Shanghai Research Center for Brain Science and Brain-Inspired Intelligence, Shanghai, China; 5 Laboratory of Perceptive Network, Shanghai Advanced Research Institute, Chinese Academy of Sciences, Shanghai, China

**Keywords:** Microtubules, Development of the nervous system

## Abstract

Tri-methylation on lysine 40 of α-tubulin (α-TubK40me3) is a recently identified post-translational modification involved in mitosis and cytokinesis. However, knowledge about α-TubK40me3 in microtubule function and post-mitotic cells remains largely incomplete. Here, we report that α-TubK40me3 is required for neuronal polarization and migration by promoting microtubule formation. α-TubK40me3 is enriched in mouse cerebral cortex during embryonic day (E)14 to E16. Knockdown of α-tubulin methyltransferase SETD2 at E14 leads to the defects in neuronal migration, which could be restored by overexpressing either a cytoplasm-localized SETD2 truncation or α-TubK40me3-mimicking mutant. Furthermore, α-TubK40me3 is preferably distributed on polymerized microtubules and potently promotes tubulin nucleation. Downregulation of α-TubK40me3 results in reduced microtubule abundance in neurites and disrupts neuronal polarization, which could be rescued by Taxol. Additionally, α-TubK40me3 is increased after losing α-tubulin K40 acetylation (α-TubK40ac) and largely rescues α-TubK40ac function. This study reveals a critical role of α-TubK40me3 in microtubule formation and neuronal development.

## Abstract (web summary)

Post-translational modifications of tubulins regulate microtubule properties and neural development. Here, the authors report that one such post-translational modification, α-TubK40me3, is required for neuronal polarization and migration by promoting microtubule formation.

## Introduction

Microtubules are highly dynamic cytoskeleton composed of α/β-tubulin heterodimers and involved in a variety of cellular processes, especially in neural development^1,2^. Microtubule functions are spatiotemporally regulated by multiple post-translational modifications (PTMs) of tubulins^2,3^. Importantly, a few PTMs, such as acetylation, detyrosination, and glutamylation, are found to be enriched in neurons and the physiological roles of these PTMs in neuronal development and functions are gaining more and more attention^3–7^. A recent study reports that in proliferative cells, α-tubulin could be tri-methylated at lysine 40 (α-TubK40me3), the same residue that has been also shown undergoing acetylation, by SET domain containing 2 (SETD2)^8^, a methyltransferase responsible for tri-methylation of histone 3 lysine 36 (H3K36me3)^9,10^. α-TubK40me3 occurs on the spindle and midbody and the loss of α-TubK40me3 causes mitotic and cytokinesis defects, indicating that α-TubK40me3 contributes to genomic stability during cell division^8^. However, the functions of α-TubK40me3 in microtubule formation and post-mitotic cells are largely unclear.

Neurons are post-mitotic cells rich in microtubules. During the development of central nervous system (CNS), formation of the six-layered structure of cerebral cortex is a tightly regulated process which is essential for the establishment of proper neuronal circuits and brain functions^11^. Projection neurons within the cerebral cortex are derived from neuronal progenitor cells (NPCs) in the ventricular zone (VZ) and subventricular zone (SVZ), and then radically migrate along the basal processes of radial glial progenitors (RGPs) to their final destinations in the cortical plate (CP)^11,12^. To initiate the radial migration, new-born projection neurons undergo morphological transition from multipolar stage to bipolar stage in the intermediate zone (IZ)^13,14^. Then, bipolar neurons extend their leading processes to the outer surface of cerebral cortex, translocate their nucleus into the leading processes and retract the trailing processes^11,12^. Recent studies are providing more insights into the importance of tubulin PTMs in cortical development. For example, impairment of α-tubulin acetylation by knocking down the acetyltransferase MEC-17 causes migratory defects in the cortical projection neurons and interneurons, and perturbs the multipolar-to-unipolar/bipolar transition of projection neurons in the IZ region^15^; Downregulation of vasohibins, the tubulin carboxypeptidases that generate detyrosinated α-tubulin, affects neuronal differentiation and radial migration of new-born cortical neurons^16^.

In this study, using a specific homemade antibody against α-TubK40me3, we show that α-TubK40me3 is enriched in NPCs and neurons from embryonic day 14 (E14) to E16 in cerebral cortex. In utero knockdown of SETD2 at E14 leads to impaired morphological transition and thereby the migration of cortical neurons, which could be restored by overexpressing NES-SETD2(1469-1724), a cytoplasm-localized SETD2 truncation retaining enzyme activity or α-tubulin^K40F^, a tri-methylation mimicking mutant of α-tubulin. Importantly, α-TubK40me3 is preferably distributed on polymerized microtubules in cells, and in vitro microtubule reconstitution assays shows that α-TubK40me3 could potently promote tubulin nucleation and slightly facilitate microtubule growth. Consistently, the microtubule abundance that is reflected by the number of EB3 comets and quantified under electron microscopy, is reduced in the neurites of cultured neurons upon downregulation of α-TubK40me3. Furthermore, promoting tubulin nucleation using a low concentration of Taxol could rescue the defects of neuronal polarization caused by impaired α-TubK40me3. Interestingly, the duration of high-level α-TubK40me3 in the developing cerebral cortex is extended in MEC-17 knockout mice, and the impaired morphological transition and migration of α-TubK40ac-deficient neurons could be largely restored by overexpressing NES-SETD2(1469-1724) and α-tubulin^K40F^, implying a compensatory effect by α-TubK40me3. All together, these results suggest that α-TubK40me3 plays an essential role in the polarization and migration of cortical neurons by promoting tubulin nucleation and controlling microtubule formation.

## Results

### A high level of α-TubK40me3 occurs during E14 to E16 in the cerebral cortex

To investigate the existence of α-TubK40me3, we generated a polyclonal antibody against peptide around tri-methylated K40 of α-tubulin (Extended Data Fig. 1a). Dot blotting and immunoprecipitation showed a good recognition on tri-methylated K40 peptide but not un-methylated or mono-methylated K40 peptide, and minimal cross-reactivity with di-methylated K40 peptide and α-tubulin^K40A^ mutant (Extended Data Fig. 1b, c), confirming a high specificity of homemade α-TubK40me3 antibody. Similar to a previous report^8^, both our homemade antibody and commercial H3K36me3 antibody could recognize α-TubK40me3 in immunoprecipitation (Extended Data Fig. 1d), however, our homemade antibody could not directly detect α-TubK40me3 in immunoblotting. Immunostaining with homemade α-TubK40me3 antibody showed no cross-reactive signals at DAPI-labeled chromatin but intense signals at α-tubulin-labeled spindle and midbody (Extended Data Fig. 1e), where a large amount of tubulin nucleation and microtubule formation take place at the metaphase and telophase^17,18^. Thus, our homemade antibody is capable for specific detection of α-TubK40me3 in both immunoprecipitation and immunostaining.

**Fig. 1:**
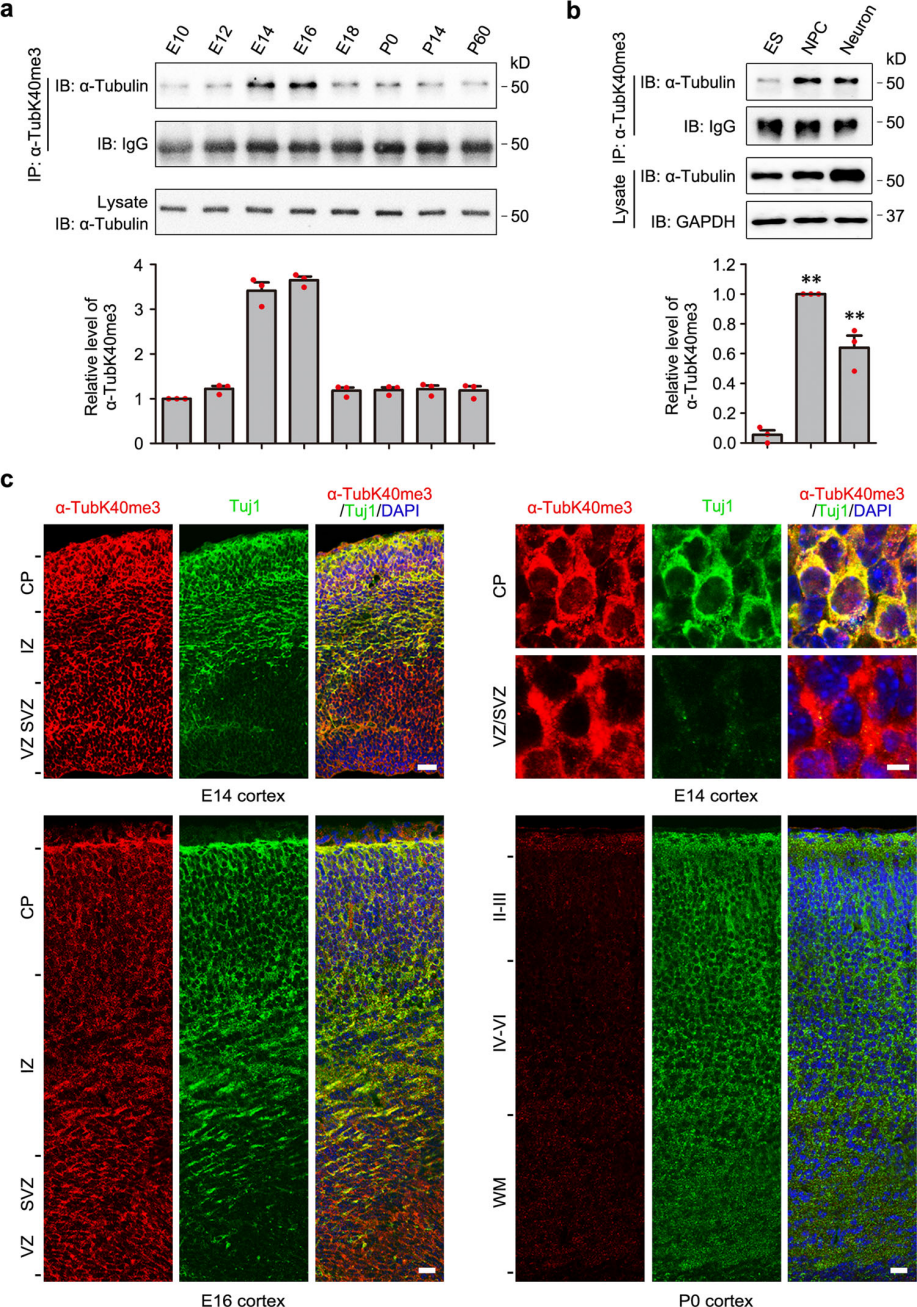
α-TubK40me3 is temporally regulated during cortical development and occurs in neural progenitor cells and neurons. **a** Immunoprecipitation by α-TubK40me3 antibody and following immunoblotting with α-tubulin showed the temporal regulation of α-TubK40me3 at different developing stages of mouse cerebral cortex. α-Tubulin and IgG served as the loading control of whole protein and antibody, respectively. The data were normalized to E10 and shown as the mean ± s.e.m. (*n* = 3 biological replicates). **b** Relatively high level of α-TubK40me3 normalized to α-tubulin existed in human NPCs and neurons but not ES cells during neuronal differentiation. GAPDH served as a loading control. The data were normalized to NPCs and shown as the mean ± s.e.m. (*n* = 3 biological replicates). Data were analyzed using paired two-tailed Student’s *t* test. **P < 0.01 versus the ES cells. **c** Immunostaining showed high level of α-TubK40me3 in the CP, IZ, and VZ/SVZ regions of mouse somatosensory cortex at E14 and E16, and large downregulation of α-TubK40me3 at P0. α-TubK40me3 was present in the NPCs of VZ/SVZ and the neurons of CP. Brain slices were stained for α-TubK40me3 (red), Tuj1 (green) and DAPI (blue) (*n* = 3 biological replicates). Scale bar: 25 μm for the brain slices and 5 μm for the upper-right high magnification. Source data are provided as a Source Data file.

To investigate the potential function of α-TubK40me3 in the development of CNS, we profiled the level of α-TubK40me3 at different developmental stages of mouse cerebral cortex. Immunoprecipitation by the α-TubK40me3 antibody and the following immunoblotting with α-tubulin showed that α-tubulin was highly tri-methylated from E14 to E16 in the mouse cerebral cortex, while only relatively low level of α-TubK40me3 was detected at other stages (Fig. 1a). To exclude the possibility that the reactive epitopes were not accessible to antibody when complexed to other proteins during regular immunoprecipitation, we performed denatured immunoprecipitation to fully expose epitopes and obtained similar results (Extended Data Fig. 1f). This distribution pattern of α-TubK40me3 was also confirmed by immunoprecipitation with commercial H3K36me3 antibody and following immunoblotting with α-tubulin antibody (Extended Data Fig. 1g). The stage-specific modification strongly suggests that α-TubK40me3 plays a role in the cerebral cortex development during E14 to E16. Notably, immunoblotting of mitotic marker phosphorylated histone 3 (PH3) revealed that cell proliferation mainly occurred at E12 rather than E14 and E16 (Extended Data Fig. 1h), suggesting that high α-TubK40me3 during E14 to E16 is involved in proliferation-independent events of cortical development. Immunoblotting of a neuron-specific β-tubulin isotype Tuj1 showed abundant neurogenesis after E12 (Extended Data Fig. 1i), raising the possibility that α-TubK40me3 is involved in the development of post-mitotic neurons.

Given that the brain tissue consists of not only neurons but also glia cells, we next detected the level of α-TubK40me3 during the induced differentiation from human embryonic stem cells (ES) to mature neurons. The relative level of α-TubK40me3 to total α-tubulin was very low in ES, increased significantly in NPCs, and was reduced and maintained at a steady level in mature neurons (Fig. 1b), suggesting an important role of α-TubK40me3 in the early stages of neuronal development. Further immunostaining on E14 and E16 brain slices in somatosensory cortex showed that α-TubK40me3 appeared in the CP and IZ, and was well co-localized with Tuj1 (Fig. 1c). At the same time, α-TubK40me3 was also observed in the VZ and SVZ (Fig. 1c), where NPCs were localized. As expected, the intensity of α-TubK40me3 in P0 cortex was relatively low (Fig. 1c). Taken together, these data reveal that α-TubK40me3 exists in NPCs and neurons, and is spatiotemporally regulated during neuronal and cortical development.

### Knockdown of SETD2 at E14 results in defects of neuronal morphology transition and thereby radial migration

**Fig. 2:**
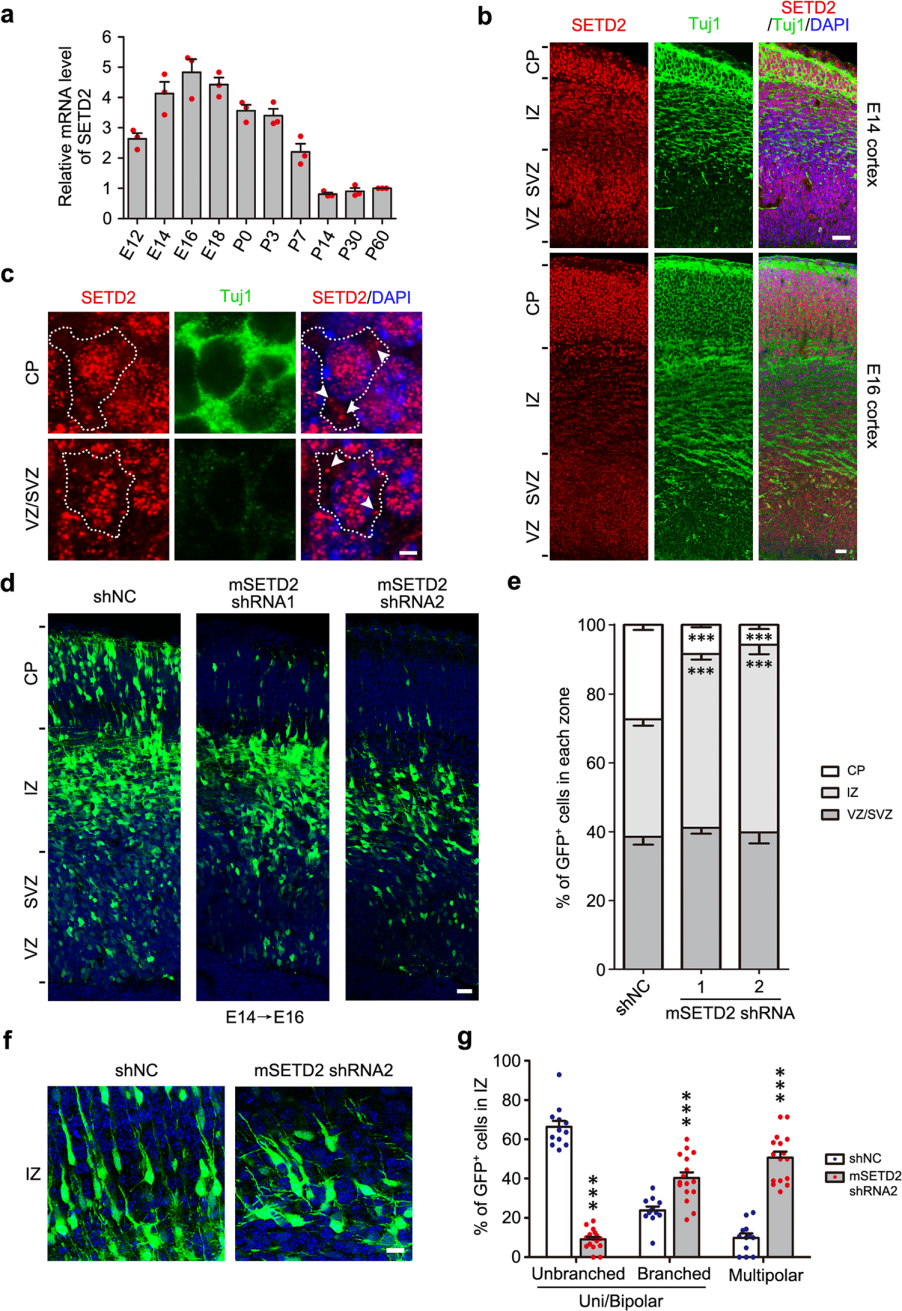
SETD2 is highly expressed during cortical development and required for neuronal migration. **a** qPCR showed that the relative level of SETD2 mRNA was high in embryonic and early post-natal mouse cerebral cortex. All data were normalized to the P60 cerebral cortex and represented as the mean ± s.e.m. (*n* = 3 biological replicates). **b** Immunostaining showed a distribution of SETD2 in CP, IZ and VZ/SVZ of mouse somatosensory cortex at E14 and E16. Brain slices were stained for SETD2 (red), Tuj1 (green) and DAPI (blue) (*n* = 3 biological replicates). Scale bar: 25 μm. **c** SETD2 was localized in the NPCs of VZ/SVZ and the neurons of CP at E14 cerebral cortex. Dashed line indicated the lineament of cell and arrowheads indicated the SETD2 puncta in the cytoplasm. Brain slices were stained for SETD2 (red), Tuj1 (green) and DAPI (blue) (*n* = 3 biological replicates). Scale bar: 2.5 μm. **d** Representative images of coronal brain sections in somatosensory cortex at E16 showed the distribution of GFP^+^ cells (green) electroporated with GFP reporter as well as control shRNA (shNC), mSETD2 shRNA1 or mSETD2 shRNA2 at E14. Sections were stained for DAPI (blue). Scale bar: 25 μm. **e** Quantitative analysis of **d** showed that the percentage of GFP^+^ cells in IZ was significantly increased after SETD2 knockdown. All data were shown as the mean ± s.e.m. (*n* = 17, 10, 11, respectively). Data were analyzed using two-way ANOVA with Bonferroni’s post-hoc test. ***P < 0.001 versus shNC. **f** Representative images of shNC and shRNA2-expressing neurons (green) at E16 in the IZ. Brain slices were stained for DAPI (blue). Scale bar: 10 μm. **g** Quantitative analysis of **f** showed that the percentage of GFP^+^ neurons at the multipolar stage in IZ was significantly increased after SETD2 knockdown. All data were shown as the mean ± s.e.m. (*n* = 12, 16, respectively). Data were analyzed using two-way ANOVA with Bonferroni’s post-hoc test. ***P < 0.001 versus shNC. Source data are provided as a Source Data file.

Since SETD2 served as the methyltransferase of α-tubulin^8^, we profiled the expression pattern of SETD2 in the cerebral cortex. Quantitative real-time PCR (qPCR) detected that the mRNA level of SETD2 was continuously increased from E12 and reached a peak at E16, which was corresponding to the high level of α-TubK40me3 at E14 and E16. The SETD2 mRNA remained at a high level until P3 and afterwards was gradually decreased (Fig. 2a). Immunostaining showed that SETD2 was expressed both in the VZ/SVZ, IZ and CP of somatosensory cortex at E14 and E16 (Fig. 2b), consistent with the distribution pattern of α-TubK40me3 at the same stages (Fig. 1c). In detail, SETD2 puncta were not only localized in the nucleus but also in the cytoplasm of cells in VZ/SVZ and CP (Fig. 2c), supporting its role in catalyzing α-TubK40me3 and fulfilling the non-chromatin function in these cells.

E14-E16 is a critical period for morphological transition and radial migration of cortical neurons^19^. Then, we addressed whether the high level of α-TubK40me3 in this period was involved in the regulation of neuronal morphology and migration. The levels of SETD2 and α-TubK40me3 were dramatically decreased in Neuro-2a cells expressing short-hairpin-interfering (sh) RNAs for mouse SETD2 (mSETD2) (Extended Data Fig. 2a, b), suggesting that acute knockdown of SETD2 by shRNA is a practical strategy to study α-TubK40me3-related functions.

Using in utero electroporation (IUE), mixed plasmids containing shRNA and GFP reporter were co-transfected to radial glia progenitors in the cerebral cortex of E14 mice. Immunostaining showed that the levels of SETD2 and α-TubK40me3 were significantly decreased in GFP-positive (GFP^+^) SETD2-deficient neurons of somatosensory cortex at E16 (Extended Data Fig. 2c–f). The level of H3K36me3 was also reduced in GFP^+^ SETD2-deficient neurons though the extent of reduction was smaller than α-TubK40me3 (Extended Data Fig. 2e, f). The distribution of GFP^+^ cells in somatosensory cortex, which was divided into CP, IZ, and VZ/SVZ, was analyzed at E16 (Fig. 2d). Approximate 27% of GFP^+^ control cells expressing control shRNA (shNC) migrated into CP, but less than 10% of GFP^+^ SETD2-deficient cells expressing mSETD2 shRNA1 or shRNA2 were able to migrate into CP and most of them were arrested in IZ (Fig. 2d, e). Most SETD2-deficient cells in IZ exhibited highly branched leading processes and multipolar morphology, whereas most control cells in IZ were bipolar with one unbranched leading process (Fig. 2f, g). The percentage of GFP^+^ cells in VZ/SVZ showed no difference between control and SETD2 knockdown (Fig. 2e), neither the percentage of cells labeled by Ki67, a marker for the proliferative cells, PH3, a marker for the mitotic cells, or cleaved caspase 3, a marker for the apoptotic cells (Extended Data Fig. 3a–d), indicating that proliferation and apoptosis of NPCs were not affected by acute SETD2 knockdown at E14. Furthermore, we did immunostaining using anti-Nestin antibody to investigate the integrity of radial glial progenitors. The result showed that basal processes of radial glial progenitors projected properly to serve as the guide for migrating neurons in SETD2 knockdown brains (Extended Data Fig. 3e), ruling out the possibility that the impaired neuronal migration is due to the disruption of guiding tracks.

To further exclude the influence of SETD2 knockdown on NPCs, we performed IUE with doublecortin promoter-driven Cre (DCX-Cre) plasmid in *Setd2*^*flox/flox*^ mice (Extended Data Fig. 4a, b) to achieve neuron-specific deletion of SETD2. Immunostaining showed that the level of α-TubK40me3 was significantly decreased in Tuj1-positive (Tuj1^+^) neurons but not in Tuj1-negative (Tuj1^-^) NPCs (Extended Data Fig. 4c, d). Meanwhile, the same defects of neuronal migration and morphology transition were observed after DCX-Cre transfection (Extended Data Fig. 4e–h), supporting that the effects of SETD2 in these processes are neuron-intrinsic. Taken together, these data suggest that SETD2 controls neuronal morphology transition and thereby neuronal migration during development of cerebral cortex.

### Cytoplasmic-localized SETD2 truncation retaining enzyme activity and tri-methylation-mimicking α-tubulin^K40F^ rescues neuronal defects induced by SETD2 knockdown at E14

**Fig. 3:**
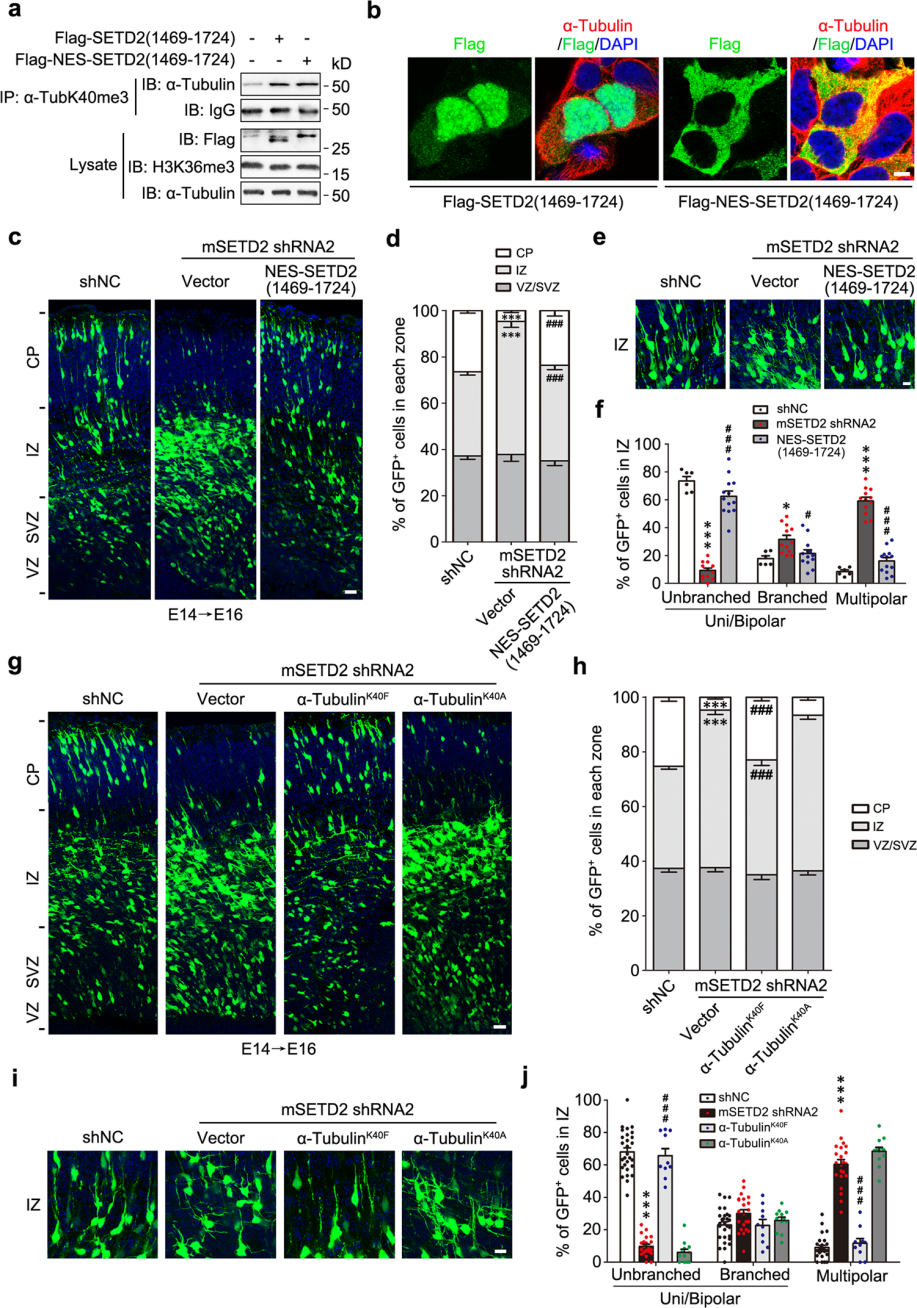
Cytoplasmic enzyme-activity-retaining SETD2 truncation and tri-methylation-mimicking mutant of α-tubulin rescue neuronal defects induced by SETD2 knockdown. **a** Immunoprecipitation by α-TubK40me3 antibody and following immunoblotting with α-tubulin showed that the level of α-TubK40me3 was increased in HEK293 cells transfected with Flag-SETD2(1469-1724) and Flag-NES-SETD2(1469-1724), while the level of H3K36me3 was not significantly changed (*n* = 3 biological replicates). **b** Immunostaining of Flag (green) and α-tubulin (red) showed the subcellular localization of Flag-SETD2(1469-1724) and Flag-NES-SETD2(1469-1724) in HEK293 cells (*n* = 3 biological replicates). Cells were stained for DAPI (blue). Scale bar: 5 μm. **c** Representative images of coronal brain sections in somatosensory cortex at E16 showed the distribution of GFP^+^ cells (green) electroporated with GFP reporter as well as shNC, mSETD2 shRNA2 or mSETD2 shRNA2 together with Flag-NES-SETD2(1469-1724) at E14. Sections were stained for DAPI (blue). Scale bar: 25 μm. **d** Quantitative analysis of **c** showed that the migration defects induced by SETD2 knockdown was rescued by expressing Flag-NES-SETD2(1469-1724) (*n* = 6, 12, 13, respectively). **e** Representative images of GFP^+^ neurons (green) in IZ. Brain slices were stained for DAPI (blue). Scale bar: 10 μm. **f** Quantitative analysis of **e** showed that the percentage of neurons at the multipolar stage in IZ was rescued by expressing Flag-NES-SETD2(1469-1724) (*n* = 6, 12, 13, respectively). **g** Representative images of coronal brain sections in somatosensory cortex at E16 showed the distribution of GFP^+^ cells (green) electroporated with GFP reporter as well as shNC, mSETD2 shRNA2 or mSETD2 shRNA2 together with Flag-α-tubulin^K40F/K40A^ at E14. Sections were stained for DAPI (blue). Scale bar: 25 μm. **h** Quantitative analysis of **g** showed that the migration defects induced by SETD2 knockdown was rescued by expressing α-tubulin^K40F^ but not α-tubulin^K40A^ (*n* = 28, 22, 10, 12, respectively). **i** Representative images of GFP^+^ neurons (green) in IZ. Brain slices were stained for DAPI (blue). Scale bar: 10 μm. **j** Quantitative analysis of **i** showed that the percentage of neurons at the multipolar stage in IZ was rescued by expressing α-tubulin^K40F^ but not α-tubulin^K40A^ (*n* = 28, 22, 10, 12, respectively). All data were shown as the mean ± s.e.m. and analyzed using two-way ANOVA with Bonferroni’s post-hoc test. *P < 0.05, ***P < 0.001 versus shNC; ^#^*P* < 0.05, ^###^*P* < 0.001 versus shRNA2. Source data are provided as a Source Data file.

Given that acute knockdown of SETD2 by shRNA at E14 reduced the level of α-TubK40me3 and H3K36me3, we designed experiments to exclusively increase the level of α-TubK40me3 to explore whether the impaired neuronal morphology and migration could be rescued. SETD2 is a multi-domain protein, in which the SET and its associated domains are responsible for binding α-tubulin and catalyzing α-TubK40me3 (Extended Data Fig. 5a–c)^8^, and the C-terminal domains containing the SRI domain is required for histone methylation in vivo^20^. Thus, we constructed a truncated SETD2(1469-1724) to selectively promote the enrichment of α-TubK40me3 but not H3K36me3. The level of α-TubK40me3 was increased in HEK293 cells transfected with SETD2(1469-1724) (Fig. 3a). However, overexpressed SETD2(1469-1724) was preferably distributed in the nucleus than the cytoplasm of HEK293 cells (Fig. 3b). To further exclude its possible effect on H3K36me3 in the nucleus, tandem nuclear export signals (NES) were added to the N-terminus of SETD2(1469-1724) to sequester the truncation in the cytoplasm (Fig. 3b), which significantly promoted the level of α-TubK40me3 without affecting H3K36me3 (Fig. 3a). Additionally, previous study reported that a SET domain mutant (R1625C) of SETD2 failed to catalyze both α-TubK40me3 and H3K36me3, and the SRI domain mutant (R2510H) was able to catalyze H3K36me3 but not α-TubK40me3 in cells^8^. The binding capability and catalytic activity of these mutants was tested in the in vitro methylation assay. Unexpectedly, R2510H mutation did not disrupt the interaction between SETD2 and α-tubulin (Extended Data Fig. 5b), and both R1625C and R2510H efficiently catalyzed α-TubK40me3 in vitro (Extended Data Fig. 5d). Thus, we chose the cytoplasmic enzyme-activity-retaining SETD2 truncation to perform the rescue experiments.

Using IUE, SETD2 shRNA and NES-SETD2(1469-1724) were co-delivered with GFP reporter into radial glia progenitors at E14. Of note, the targeting sequence of mSETD2 shRNA2 was not comprised in SETD2(1469-1724). Immunostaining showed that Flag-NES-SETD2(1469-1724) was localized in the cytoplasm of GFP^+^ cells in brain slice at E16 and restored the level of α-TubK40me3 but not H3K36me3 in GFP^+^ SETD2-deficient neurons (Extended Data Figs. 2e, f and 5e). At the same time, NES-SETD2(1469-1724) could rescue the defects of neuronal morphology transition and migration in somatosensory cortex (Fig. 3c–f), suggesting that the effects of SETD2 on cortical development during E14-E16 was dependent on its cytoplasmic methyltransferase-activity. To further confirm the contribution of α-TubK40me3 in the development of cerebral cortex, we expressed α-tubulin^K40F^, a tri-methylation-mimicking mutant by increasing the hydrophobicity and local bulkiness of distal R group of lysine^21–23^, and α-tubulin^K40A^ as a negative control in SETD2-deficient neurons (Fig. 3g). Both mutants were able to be assembled into microtubule network properly in HEK293 cells (Extended Data Fig. 5f). Immunostaining showed that the defects in neuronal morphology transition and migration caused by SETD2 knockdown were efficiently rescued by α-tubulin^K40F^, but not α-tubulin^K40A^ at E16 (Fig. 3g–j). Taken together, these data indicate that SETD2-mediated α-TubK40me3 is a critical PTM for neuronal migration in cerebral cortex during E14-E16.

### α-TubK40me3 promotes tubulin nucleation and microtubule polymerization

**Fig. 4:**
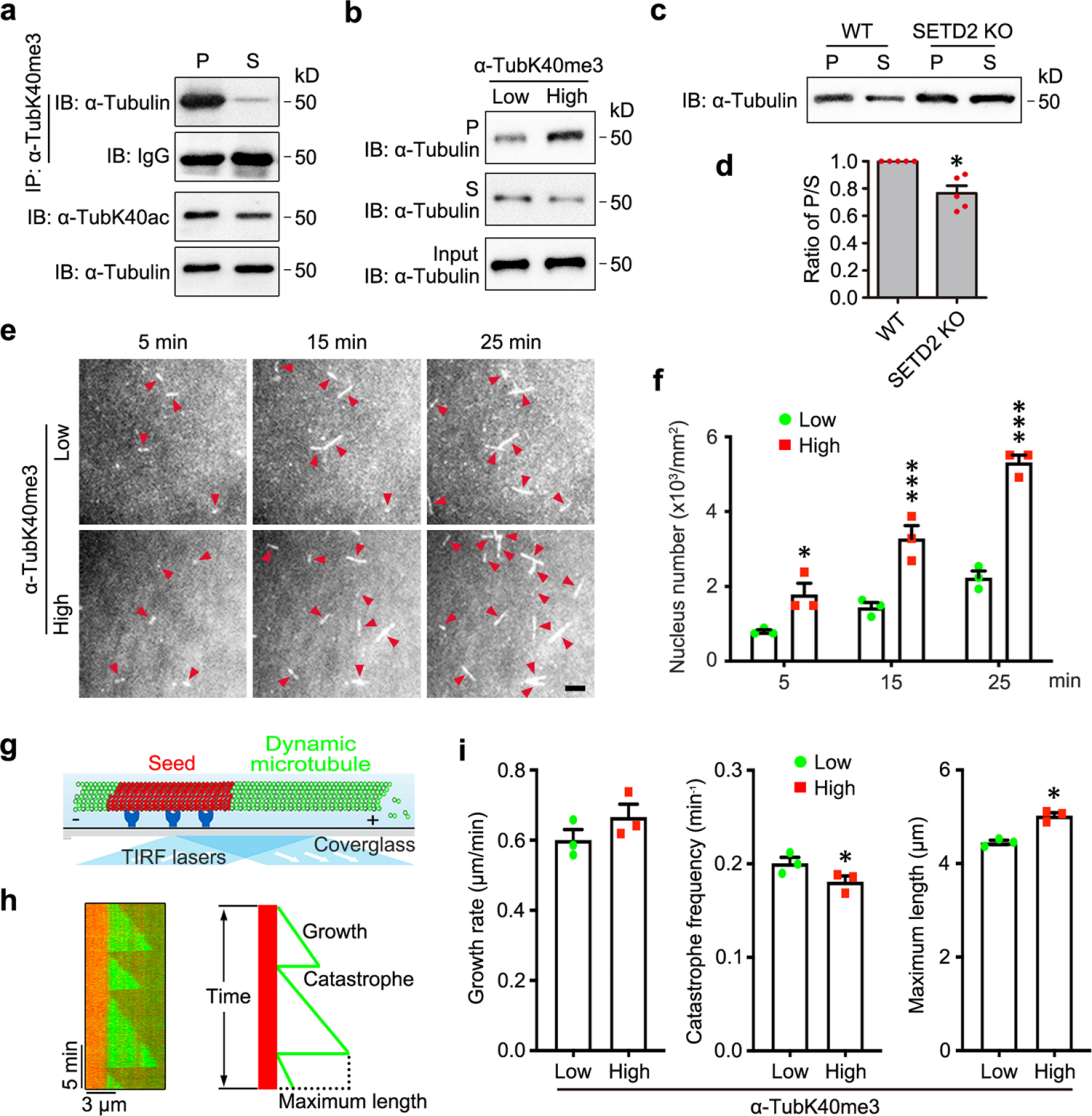
α-TubK40me3 promotes microtubule polymerization in cells and in vitro. **a** Immunoprecipitation by α-TubK40me3 antibody from soluble (S) and polymerized (P) tubulins and following immunoblotting with α-tubulin showed that α-TubK40me3 was preferably distributed on polymerized but not soluble tubulins in HEK293 cells (*n* = 3 biological replicates). **b** Immunoblotting after sedimentation assay with low or high methylation level tubulins showed that α-TubK40me3 promoted microtubule formation (*n* = 3 biological replicates). **c**, **d** Immunoblotting and quantitative analysis showed that the ratio of P/S tubulin was decreased after SETD2 knockout in HEK293 cells. All data were shown as the mean ± s.e.m. (*n* = 5 biological replicates). Data were analyzed using paired two-tailed Student’s *t* test. *P < 0.05 versus WT. **e**, **f** Representative images and quantitative analysis at 5, 15 and 25 min from in vitro tubulin nucleation assay using 20 µM low or high methylation level tubulins showed that α-TubK40me3 highly promoted tubulin nucleation. Arrowheads indicated the microtubule nucleus. All data were shown as the mean ± s.e.m. (*n* = 3 biological replicates). Data were analyzed using paired two-tailed Student’s *t* test. *P < 0.05, ***P < 0.001 versus low methylation level. Scale bar: 5 μm. **g** Schematic of the observation for in vitro microtubule reconstitution by TIRF microscopy. The red part represents the GMPCPP-stabilized microtubule seeds and the green part indicates the dynamic microtubules growing from the seeds. **h** Kymograph of dynamic microtubules growing from seeds by time-lapse imaging with TIRF microscopy (left). Schematic diagram for the parameters of microtubule dynamics (right). **i** Quantitative analysis of microtubule dynamics using 20 µM low or high methylation level tubulins showed that α-TubK40me3 slightly reduced the catastrophe frequency, which may lead to increased maximum length of microtubules. All data were shown as the mean ± s.e.m. (*n* = 3 biological replicates, low methylation level: 93 microtubules; high methylation level: 108 microtubules). Data were analyzed using paired two-tailed Student’s *t* test. *P < 0.05 versus the low methylation level. Source data are provided as a Source Data file.

The underlying mechanism of α-TubK40me3 affecting neuronal development was explored. In cells, tubulins either exist in soluble form or polymerize into microtubules. We separated soluble and polymerized tubulins in HEK293 cells and detected the level of α-TubK40me3 in these two pools. Immunoprecipitation and immunoblotting showed that α-TubK40me3 was preferably distributed on polymerized microtubules (Fig. 4a), consistent with the result that α-TubK40me3 is mainly found in spindle and midbody^8^ (Extended Data Fig. 1e). Importantly, compared with α-TubK40ac, α-TubK40me3 showed an even stronger preference for polymerized microtubules (Fig. 4a). To further clarify whether α-TubK40me3 actively promoted tubulin assembly, we first prepared highly methylated tubulin by in vitro methylation reaction using purified adult mouse brain tubulin as substrates. Original tubulin only contained a low level of α-TubK40me3 and after reaction, the methylation level of tubulin was significantly increased (Extended Data Fig. 6a–c). Separation of soluble tubulin and polymerized microtubule after in vitro microtubule assembly revealed that methylated tubulin was able to generate more microtubule (Fig. 4b). Furthermore, SETD2 knockout significantly decreased the level of polymerized microtubules and resulted in the reduction of α-TubK40ac-labeled stable microtubules in HEK293 cells (Fig. 4c, d and Extended Data Fig. 6d). Thus, α-TubK40me3 favors microtubule formation.

Previous study proposed that microtubule formation consists of two main steps: tubulin nucleation and microtubule elongation. Tubulin nucleation is the early step to produce the seeds, and microtubule elongation is the late step for tubulin dimers to add on the existing seeds^24^. We conducted in vitro tubulin nucleation to explore if α-TubK40me3 affected tubulin nucleation. Time-lapse total internal reflection fluorescence (TIRF) imaging showed that the number of short microtubules was gradually increased in a time-dependent manner. Notably, the number of short microtubules was largely increased in the chamber of tubulins with the high methylation level compared to the low one (Fig. 4e, f), suggesting that α-TubK40me3 significantly improves tubulin nucleation. Furthermore, in vitro microtubule reconstitution assay was performed using tubulins with low or high methylation level to study the role of α-TubK40me3 in microtubule elongation (Fig. 4g, h). Time-lapse TIRF imaging and quantitative data showed that the microtubule catastrophe frequency was decreased but the microtubule growth rate was not significantly changed, leading to increased maximum length of microtubules in the chamber of tubulins with high methylation level (Fig. 4i). Taken together, these data support that α-TubK40me3 potently promotes tubulin nucleation and slightly reduces microtubule dynamics, thereby facilitates microtubule formation.

### α-TubK40me3 promotes microtubule formation in neurons

**Fig. 5:**
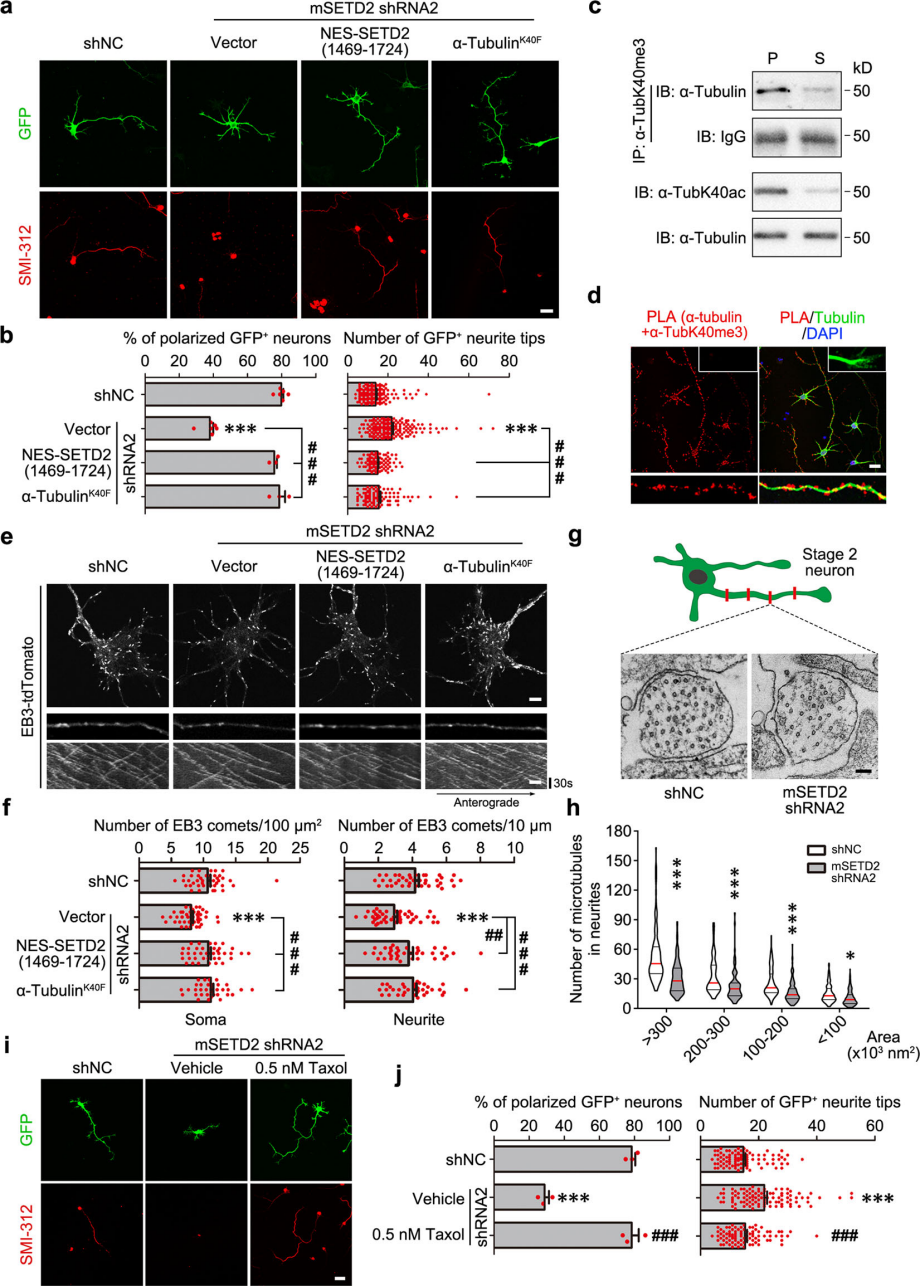
Knockdown of SETD2 results in reduced microtubule number and polarization defects in cultured neurons. **a** Representative images of hippocampal neurons cultured for 3 days from E14 mice. The neurons were electroporated with GFP reporter as well as shNC, mSETD2 shRNA2 or mSETD2 shRNA2 together with Flag-NES-SETD2(1469-1724) or α-tubulin^K40F^. Fixed neurons were stained for GFP (green) and SMI-312 (red). Scale bar: 25 μm. **b** Quantitative analysis of **a** showed that the percentage of polarized GFP^+^ neurons was significantly decreased and the total number of neurite tips including all neurites and their protrusions was increased after SETD2 knockdown. These defects were rescued by expressing Flag-NES-SETD2(1469-1724) or α-tubulin^K40F^. All data were shown as the mean ± s.e.m. (168, 174, 99 and 92 neurons respectively collected from 3 biological replicates with similar results). Data were analyzed using unpaired two-tailed Student’s *t* test. ***P < 0.001 versus shNC; ^###^*P* < 0.001 versus shRNA2. **c** Immunoprecipitation by α-TubK40me3 antibody from soluble (S) and polymerized (P) tubulins and following immunoblotting with α-tubulin showed that α-TubK40me3 was preferably distributed on polymerized but not soluble tubulins in cultured E14 cortical neurons (*n* = 3 biological replicates). **d** PLA signals (red) representing α-TubK40me3 was present in soma and axon shaft but not grow cone of cultured hippocampal neurons at DIV 3. Neurons were stained for tubulin (green) and DAPI (blue) after PLA reaction (*n* = 3 biological replicates). Scale bar: 25 μm. **e** Representative images and kymographs of EB3-tdTomato in E14 hippocampal neurons at DIV 3. The neurons were electroporated with EB3-tdTomato plasmid as well as shNC, mSETD2 shRNA2 or mSETD2 shRNA2 together with Flag-NES-SETD2(1469-1724) or α-tubulin^K40F^. Scale bar: 5 μm for the soma and 2.5 μm for the kymograph. **f** Quantitative analysis of **e** showed that the numbers of EB3 comets within both soma and neurites were significantly decreased after SETD2 knockdown. These defects were rescued by expressing Flag-NES-SETD2(1469-1724) or α-tubulin^K40F^. All data were shown as the mean ± s.e.m. (41, 44, 39 and 35 neurons respectively collected from 3 biological replicates with similar results). Data were analyzed using unpaired two-tailed Student’s *t* test. ***P < 0.001 versus shNC; ^##^*P* < 0.01, ^###^*P* < 0.001 versus shRNA2. **g** Representative TEM images of microtubules in the neurites of E14 hippocampal neurons at DIV 2. shNC or mSETD2 shRNA2 was delivered into these neurons by lentivirus. Scale bar: 100 nm. **h** Quantitative analysis of **g** showed that the number of microtubules in neurites at different cross-sectional areas was significantly decreased after SETD2 knockdown. All data were shown as the violin plots (>500 neurites each from 2 trials). Red line indicated the median and black line indicated the quartiles. Data were analyzed using unpaired two-tailed Student’s *t* test. *P < 0.05, ***P < 0.001 versus shNC. **i** Representative images of E14 hippocampal neurons at DIV3. The neurons were electroporated with GFP reporter as well as shNC or mSETD2 shRNA2. 0.5 nM Taxol was applied to neurons 24 h after plating. Fixed neurons were stained for GFP (green) and SMI-312 (red). Scale bar: 25 μm. **j** Quantitative analysis of **i** showed that the defects in neuronal polarization and arborization were rescued by applying 0.5 nM Taxol. All data were shown as the mean ± s.e.m. (91, 99 and 88 neurons respectively collected from 3 biological replicates with similar results). Data were analyzed using unpaired Student’s *t* test. ***P < 0.001 versus shNC; ^###^*P* < 0.001 versus shRNA2. Source data are provided as a Source Data file.

To explore whether α-TubK40me3 affected microtubule formation in neurons, we used cultured system and first detected the expression of SETD2 and α-TubK40me3 in neurons. The level of α-TubK40me3 in cultured cortical neurons was continuously increased during development (Extended Data Fig. 7a). Immunostaining showed that SETD2 puncta were localized in the nucleus, soma and neurites of neurons, and acute knockdown by shRNA significantly reduced SETD2 in neurons (Extended Data Fig. 7b). Since cultured hippocampal neurons displayed distinct polarized morphology, they were isolated from E14 mice and transfected with mSETD2 shRNA and GFP reporter by electroporation to examine whether downregulation of α-TubK40me3 impaired the neuronal morphogenesis. The majority of control neurons was polarized and developed one distinct and SMI-312^+^ axon at day in vitro (DIV) 3 (Fig. 5a). In contrast, the polarization percentage was significantly decreased in SETD2 knockdown neurons, most of which were multipolar with neurites but had no SMI-312^+^ axon (Fig. 5a, b). Additionally, the number of neurite tips, include all neurites and their protrusions, was largely increased in SETD2 knockdown neurons (Fig. 5b). These defects of neuronal polarization and arborization could be efficiently rescued by NES-SETD2(1469-1724) and α-tubulin^K40F^ (Fig. 5a, b). Thus, α-TubK40me3 also plays critical roles in the development of cultured neurons, similar to that in cerebral cortex.

Then, we examined the regulation of neuronal microtubule formation by α-TubK40me3. Firstly, we compared the distribution of α-TubK40me3 in soluble tubulin and polymerized microtubule in cultured neurons. The result also showed that α-TubK40me3 was preferably distributed on polymerized microtubules in neurons (Fig. 5c). The subcellular localization of α-TubK40me3 was detected in neurons using proximity ligation assay (PLA). The PLA signals with only α-TubK40me3 antibody and α-tubulin/β-tubulin antibodies were regarded as the negative and positive control, respectively (Extended Data Fig. 7c). The PLA signals representing α-TubK40me3 with α-TubK40me3/α-tubulin antibodies were present in the soma, dendrites and axon shaft of cultured hippocampal neurons, but not in the growth cone (Fig. 5d), where microtubules were relatively dynamic, in line with the biochemical data that α-TubK40me3 preferentially distributed on polymerized and stable microtubules (Figs. 4a and 5c). As expected, the PLA signals in cultured neurons were decreased after SETD2 knockdown both in cell body and neurites (Extended Data Fig. 7d, e), supporting the efficiency of SETD2 knockdown on α-TubK40me3 and the specificity of PLA signals representing α-TubK40me3. We further expressed EB3-tdTomato to label microtubule plus-ends and perform time-lapse imaging in control and SETD2-deficient hippocampal neurons at DIV 3. Quantitative data showed that the number of EB3 comets was significantly decreased both in the soma and neurites after SETD2 knockdown, and this reduction was rescued by the expression of NES-SETD2(1469-1724) and α-tubulin^K40F^ (Fig. 5e, f). However, the velocity, growth lifetime, catastrophe frequency and percentage of anterograde movement in EB3 comets were not changed in the neurites of SETD2-deficient neurons (Extended Data Fig. 7f), suggesting that α-TubK40me3 does not affect microtubule plus-end dynamics and orientation. Further detection with electron microscopy showed that the number of microtubules at different segments of neurites was significantly decreased in cultured hippocampal neurons at DIV 2 after SETD2 knockdown by lentivirus (Fig. 5g, h and Extended Data Fig. 7g). Since Taxol promotes tubulin nucleation and stabilizes microtubules^25,26^, we wonder whether it could regulate the neuronal polarization and arborization induced by SETD2 deficiency. As expected, the defects of neuronal polarization and arborization caused by SETD2 knockdown were efficiently rescued by 0.5 nM Taxol (Fig. 5i, j). Taken together, these data support that α-TubK40me3 promotes microtubule formation and maintains microtubule number in neurons.

### α-TubK40me3 is able to rescue the defects of radial migration and morphological transition in α-TubK40ac-deficient cortical neurons

**Fig. 6:**
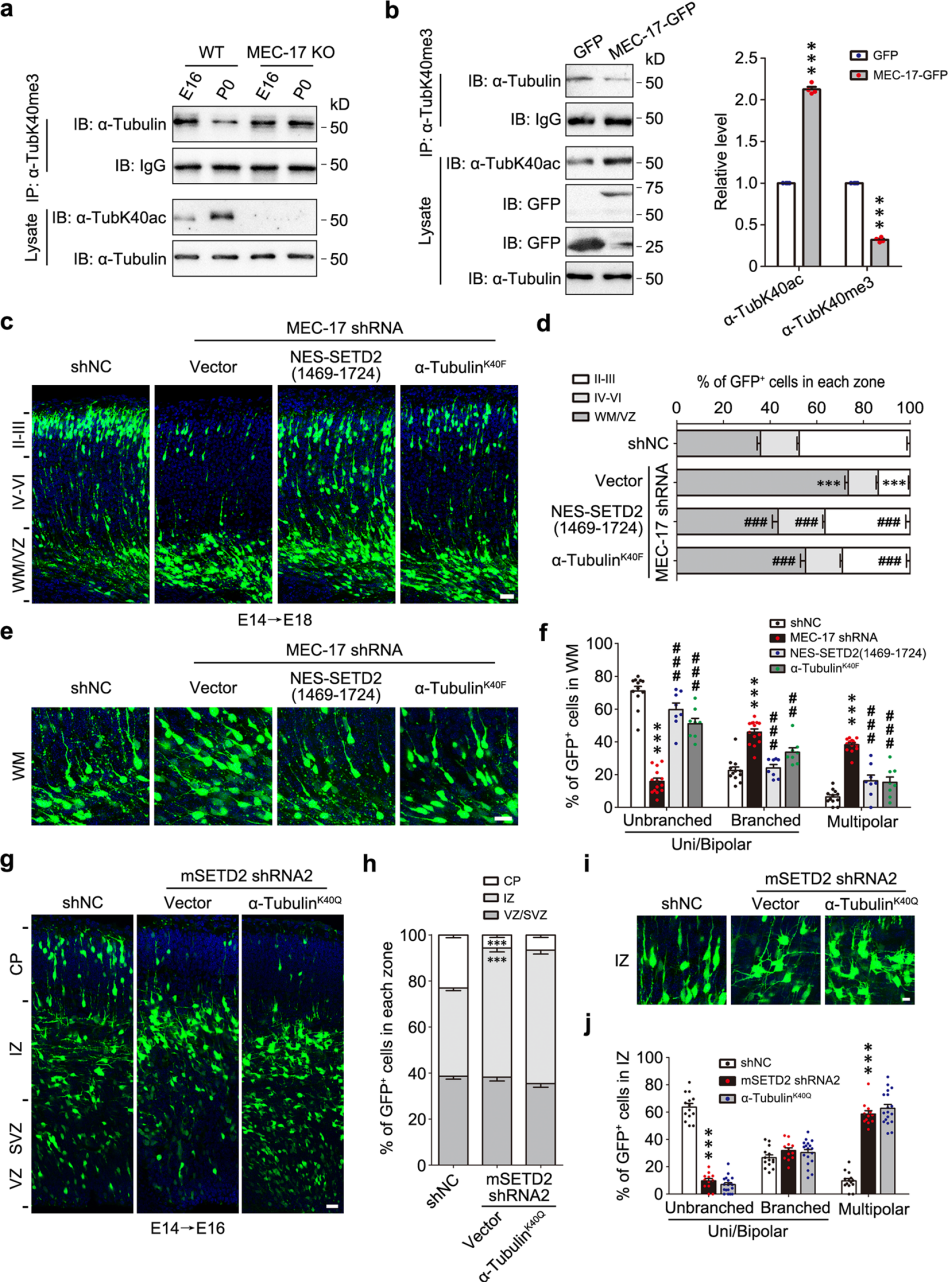
α-TubK40me3 is increased after MEC-17 knockout and able to rescue the defects of α-TubK40ac-deficient neurons. **a** Immunoprecipitation by α-TubK40me3 antibody and following immunoblotting with α-tubulin showed that the level of α-TubK40me3 was not decreased at P0 in the cerebral cortex of MEC-17 knockout mice (*n* = 3 biological replicates). **b** Immunoprecipitation and quantitative analysis showed that the level of α-TubK40me3 was significantly reduced after overexpression of MEC-17 in HEK293 cells. All data were shown as the mean ± s.e.m. (*n* = 4 biological replicates). Data were analyzed using paired two-tailed Student’s *t* test. ***P < 0.001 versus GFP. **c** Representative images of coronal brain sections in somatosensory cortex at E18 showed the distribution of GFP^+^ cells (green) electroporated with GFP reporter as well as shNC, MEC-17 shRNA or MEC-17 shRNA together with NES-SETD2(1469-1724) or α-tubulin^K40F^ at E14. Sections were stained for DAPI (blue). Scale bar: 30 μm. **d** Quantitative analysis of **c** showed that the migration defects induced by MEC-17 knockdown was highly rescued by expressing either NES-SETD2(1469-1724) or α-tubulin^K40F^. All data were shown as the mean ± s.e.m. (*n* = 18, 22, 18, 14, respectively). Data were analyzed using two-way ANOVA with Bonferroni’s post-hoc test. ***P < 0.001 versus shNC; ^###^*P* < 0.001 versus MEC-17 shRNA. **e** Representative images of GFP^+^ neurons (green) in white matter (WM). Brain slices were stained for DAPI (blue). Scale bar: 20 μm. **f** Quantitative analysis of **e** showed that the percentage of GFP^+^ neurons at the multipolar and branched uni/bipolar stage was largely rescued by expressing either NES-SETD2(1469-1724) or α-tubulin^K40F^. All data were shown as the mean ± s.e.m. (*n* = 12, 14, 8, 8, respectively). Data were analyzed using two-way ANOVA with Bonferroni’s post-hoc test. ***P < 0.001 versus shNC; ^##^*P* < 0.01, ^###^*P* < 0.001 versus MEC-17 shRNA. **g** Representative images of coronal brain sections in somatosensory cortex at E16 showed the distribution of GFP^+^ cells (green) electroporated with GFP reporter as well as shNC, mSETD2 shRNA2 or mSETD2 shRNA2 together with Flag-α-tubulin^K40Q^ at E14. Sections were stained for DAPI (blue). Scale bar: 25 μm. **h** Quantitative analysis of **g** showed that the migration defects induced by SETD2 knockdown was not rescued by expressing α-tubulin^K40Q^. All data were shown as the mean ± s.e.m. (*n* = 16, 12, 18, respectively). Data were analyzed using two-way ANOVA with Bonferroni’s post-hoc test. ***P < 0.001 versus shNC. **i** Representative images of GFP^+^ neurons (green) in IZ. Brain slices were stained for DAPI (blue). Scale bar: 10 μm. **j** Quantitative analysis of **i** showed that the percentage of GFP^+^ neurons at the multipolar stage was not rescued by expressing α-tubulin^K40Q^. All data were shown as the mean ± s.e.m. (*n* = 14, 12, 18, respectively). Data were analyzed using two-way ANOVA with Bonferroni’s post-hoc test. ***P < 0.001 versus shNC. Source data are provided as a Source Data file.

Since α-TubK40me3 and α-TubK40ac occurs at the same lysine residue of α-tubulin, a crosstalk possibly exists between them. Despite the fact that SETD2 was highly expressed in cerebral cortex throughout embryonic stages (Fig. 2a), the high level of α-TubK40me3 only occurred during E14-E16 and rapidly decreased at E18 (Fig. 1a). qPCR showed that the mRNA level of MEC-17 and α-tubulin deacetylase HDAC6 was gradually increased from E14 to E16 and reached the peak at E18 and P0, respectively (Extended Data Fig. 8a), indicating a multifactorial regulation of α-TubK40ac at late embryonic stages in cerebral cortex. These results raise a possibility that the increased α-TubK40ac after E16 occupies lots of K40 sites and results in the reduction of α-TubK40me3. To test this hypothesis, we examined the level of α-TubK40me3 in the cerebral cortex of MEC-17 knockout mice at E16 and P0. In wildtype (WT) mice, the level of α-TubK40me3 was reduced at P0, which was accompanied with increased α-TubK40ac, while α-TubK40me3 was no longer reduced at P0 and even maintained at a high level until adult in MEC-17 knockout mice (Fig. 6a and Extended Data Fig. 8b–d). Moreover, upregulating α-TubK40ac by MEC-17 overexpression in HEK293 cells significantly reduced the level of α-TubK40me3 (Fig. 6b). Thus, increased α-TubK40ac causes decreased α-TubK40me3 during cortical development, conversely loss of α-TubK40ac results in enhanced α-TubK40me3.

According to our previous study, reduced α-TubK40ac by MEC-17 knockdown leads to impaired migration of cortical neurons^15^. However, the neuronal defects of radial migration observed in acute knockdown experiments seemed mild-to-null in MEC-17 knockout mice (Extended Data Fig. 8e, f). Considering the role of α-TubK40me3 in microtubule formation and neuronal development, an increased level of α-TubK40me3 may compensate for the defects of α-TubK40ac loss in MEC-17 knockout mice. We adopted MEC-17 shRNA to downregulate the level of α-TubK40ac (Extended Data Fig. 8g, h). After IUE, the defects in radial migration and morphological transition of cortical neurons by MEC-17 knockdown at E14 were largely rescued by increased level of α-TubK40me3, which was generated by the expression of either NES-SETD2(1469-1724) or α-tubulin^K40F^ (Fig. 6c–f), indicating a compensatory effect of α-TubK40me3 for α-TubK40ac. Conversely, the neuronal defects by SETD2 knockdown at E14 were not rescued by expressing α-tubulin^K40Q^ (Fig. 6g–j), a widely used variant to mimic α-TubK40ac^7,15^, similar to the effect of delivering α-tubulin^K40A^ (Fig. 3g–j). In addition, α-tubulin^K40Q^ was able to assemble into microtubule network properly in HEK293 cells (Extended Data Fig. 5f). Taken together, these data suggest that α-TubK40me3 is able to rescue the defects of radial migration and morphological transition of cortical neurons caused by α-TubK40ac deficiency.

## Discussion

**Fig. 7:**
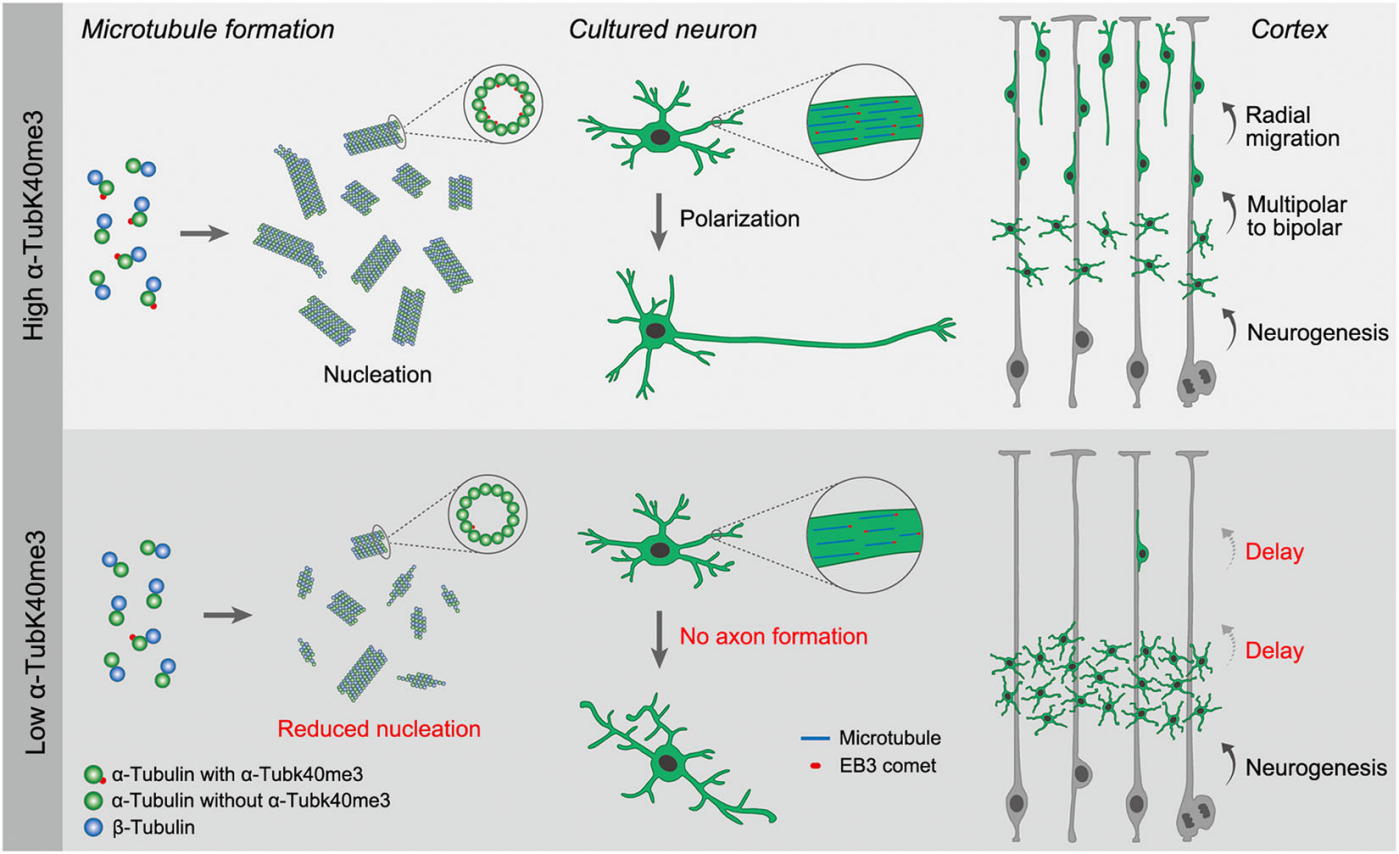
A proposed model for the role of α-TubK40me3 in regulating microtubule formation as well as neuronal polarization and migration. In cerebral cortex, developmentally upregulated α-TubK40me3 facilitates tubulin nucleation and promotes the neuronal exit from multipolar stage, thereby initiating the radial migration. In the absence of α-TubK40me3, tubulin nucleation is not easy to form and the number of microtubules is reduced in neurons, leading to defects in neuronal polarization and migration.

The spatiotemporal regulation of tubulin PTMs is crucial for modulating microtubule properties and neural development. Here, we found that SETD2-mediated α-TubK40me3 promotes microtubule formation and is required for proper neuronal development. In developing cortical neurons, α-TubK40me3 promotes microtubule formation and facilitates the morphological transition and radial migration during E14-16. Downregulation of α-TubK40me3 results in a reduction of microtubule number and causes defects of neuronal polarization (Fig. 7). These findings greatly expand our knowledge about the functions and mechanisms of α-tubulin methylation in post-mitotic cells.

### α-TubK40me3 promotes microtubule formation

The “tubulin code” hypothesis, as an analogy to the concept of “histone code”, has been proposed to describe how various PTMs of tubulins regulate microtubule-associated cellular processes^3^. SETD2-mediated α-TubK40me3 is a recently identified tubulin code^8^. Unlike most tubulin C-terminal PTMs, tri-methylation and acetylation on lysine 40 occur in the lumen of microtubules. Since lysine 40 is localized in one of the two loops that form the lateral contact of α/β-tubulin heterodimers^27^, recent studies propose a model that α-TubK40ac could weaken the lateral interaction between protofilaments and protect long-lived microtubules from mechanical breakage^24,28^. Notably, the existence of α-TubK40me3 on the same residue provides an additional possibility to regulate microtubule properties. Unlike acetylation that neutralizes the positive charge of lysine, methylation does not alter the charge on lysine and may operate through non-electrostatic mechanisms such as hydrophobic interaction^29^. The lateral interface between neighboring protofilaments is described as the “lock and key” model, which includes the M-loop (S7-H9 loop) on one side, sandwiched by the H1-S2 loop and H2-S3 loop on the other side^30^. α-TubK40me3 on the H1-S2 loop may change its conformation and bulkiness or increase the hydrophobic interaction to further lock the M-loop, resulting in strengthened lateral interaction of α/β-tubulin heterodimers and enhanced tubulin nucleation.

In cells, we found that α-TubK40me3 was enriched in specialized regions consisting of copious microtubules such as spindle, midbody and shaft of neuronal axons, where extensive microtubule formation takes place^1,17^. Most importantly, the time-lapse imaging of EB3 comets and electron microscopy showed that loss of α-TubK40me3 resulted in a decreased number of microtubules without affecting the growth velocity and direction of growing microtubules in neurites, suggesting an essential role of α-TubK40me3 in microtubule formation but not elongation in neurons. These findings indicate the requirement of α-TubK40me3 for microtubule formation in post-mitotic cells.

### α-TubK40me3 is required for neuronal polarization and migration

In the present study, the distinct expression pattern of SETD2, which is defined as a dual-functional methyltransferase for histone and α-tubulin, was observed in the VZ/SVZ, IZ, and CP regions of developing cerebral cortex. Previous study reveals that homozygous disruption of *Setd2* leads to embryonic lethality at E10.5–E11.5, which is accompanied by defects of neural development including unclosed neural tube and forebrain hypoplasia^10^. Mutations in SETD2 have been linked to brain disorders such as intellectual disabilities and autism spectrum disorders^31,32^. SETD2-mediated H3K36me3 is a well-characterized epigenetic marker associated with active gene transcription and plays important roles in neural development^33^. Acute knockdown of SETD2 by shRNAs led to severe defects in neuronal development, along with significantly disrupted α-TubK40me3 and less affected H3K36me3. The smaller impact on H3K36me3 level after acute SETD2 knockdown might be due to the relatively higher abundance of SETD2 in nucleus or relatively low turnover rate of H3K36me3-labeled histones^34^. As expected, the role of SETD2-mediated α-TubK40me3 in neuronal development is also supported by the evidence that the defects in neuronal polarization and migration by SETD2 knockdown could be efficiently rescued by a cytoplasmic-localized truncation of SETD2 retaining enzyme activity as well as the α-TubK40me3-mimicking mutant.

Both immunoblotting and immunostaining showed that the level of α-TubK40me3 was dramatically increased during E14-E16 in the developing cerebral cortex, which is a critical period for neuronal migration^19^. Local polymerization and stabilization of microtubules is required for the leading process formation and morphological transition from multipolar stage to bipolar stage to initiate the radial migration^13,35^. Previous study also reveals that the nucleation of non-centrosomal microtubules in the axon is required for neuronal polarization^36^. In the present study, the reduced microtubule number in the neurites after SETD2 knockdown imply an essential role of α-TubK40me3 in neuronal microtubule formation. Further evidence that application of 0.5 nM Taxol to promote tubulin nucleation and stabilize microtubules completely rescues the defect of neuronal polarization caused by SETD2 knockdown finally supports an importance of α-TubK40me3 in facilitating microtubule formation during neuronal development.

### A crosstalk exists between α-TubK40me3 and α-TubK40ac

The temporal regulation of α-TubK40me3 in cerebral cortex was not corelated to the expression profile of SETD2, indicating that additional regulatory mechanisms might exist. Despite the highest mRNA level of α-tubulin deacetylase HDAC6 at P0, the level of α-TubK40ac was significantly increased from E16 to P0, implying that the post-transcriptional mechanisms, probably PTMs and subcellular localization of relevant enzymes, are also involved in regulating α-TubK40ac level. Our study suggests that increased level of α-TubK40ac after E16 in cerebral cortex results in the downregulation of α-TubK40me3 possibly due to the competition for the K40 site. The preponderance of α-TubK40ac in this competition may be further evidenced by relatively higher expression level or catalytic efficiency of MEC-17 than SETD2 in cytoplasm. Furthermore, the spatiotemporal distribution and related mechanisms of α-TubK40me3 and α-TubK40ac in various microtubule-associated processes remain to be explored.

Due to the catalytic preference of MEC-17 for microtubules over free tubulins, α-TubK40ac mostly occurs on post-assembly microtubules and is widely thought as a marker for stable microtubules^37^, while α-TubK40ac per se does not affect assembly and disassembly of microtubules^38^. Given that α-TubK40ac weakens the lateral interaction between protofilaments^24,28^ and dampens microtubule plus-end dynamics^7^, these results imply that α-TubK40ac functions through protecting long-lived microtubules from mechanical breakage and regulating plus-end dynamics. In contrast, polymerization of microtubules is not required for α-TubK40me3 catalysis by SETD2, because SETD2 methylates both soluble tubulins and polymerized microtubules^8^. In the present study, we found that methylated tubulins are more prone to nucleate and promote microtubule formation, leading to the fact that α-TubK40me3 are mostly detected on polymerized microtubules. These findings support that α-TubK40me3 and α-TubK40ac work collaboratively to ensure microtubule functions by promoting tubulin nucleation and protecting existent microtubules, respectively. Since the defects in radial migration and morphological transition of cortical neurons by MEC-17 knockdown were largely rescued by increased α-TubK40me3, we propose that the upregulation of α-TubK40me3 may compensate for the defects of neuronal development in MEC-17 knockout mice.

Our previous study suggests that α-TubK40ac restricts axon overbranching and overgrowth by dampening microtubule plus-end dynamics in neurons^7^. The axon overbranching was observed in the cerebral cortex and cultured neurons of MEC-17 knockout mice, despite under both conditions the level of α-TubK40me3 was maintained at a high level, indicating that increased α-TubK40me3 does not compensate for the overbranching defect due to loss of α-TubK40ac. More importantly, the axon overbranching was efficiently rescued by overexpression of MEC-17 and α-TubK40ac-mimicking α-tubulin^K40Q^. Mechanistically, loss of α-TubK40ac led to hyperdynamics in microtubule plus-ends, including increased growth rate and catastrophe frequency, and suppression of microtubule plus-end hyperdynamics could rescue axon overbranching in MEC-17-deficient neurons^7^. However, α-TubK40me3 mainly promotes microtubule formation without altering microtubule plus-end dynamics in neurons. Thus, the enhanced axon outgrowth and branching in MEC-17 knockout neurons is derived from loss of α-TubK40ac itself. Therefore, α-TubK40me3 is able to rescue the defects of morphological transition and migration but not axon overbranching in MEC-17-deficient neurons.

## Methods

### Plasmids

The expression constructs for human SETD2 (NM_014159) 1469-1724 and 1392-2564 amino acids were subcloned from SETD2-GFP plasmid (Addgene), and the PCR-amplified fragment linked with Flag-coding sequence was inserted into pCAG-IRES-eGFP vector for overexpression or pGEX-4T-1 vector for protein purification. Tandem NES of mouse STAT1 (SLAAEFRHLQLK) was added to the N-terminus of SETD2 truncation. The expression construct for rat TUBA1A (NM_022298.1), of which amino acid sequence is totally conserved between rat and mouse, was modified from previously described plasmids^15^ by inserting Flag tag into the N-terminus of α-tubulin. The R1625C and R2510H mutations of SETD2, and the K40F, K40Q and K40A mutations of TUBA1A were generated by PCR-mediated mutagenesis kit (Toyobo) and verified by sequencing. The end binding protein 3 (EB3) (NM_001007656.1) was cloned from the cDNA of rat dorsal root ganglion into the pCDH-CMV vector with tdTomato tag on the C-terminus. All shRNA constructs were inserted into the pGPU6-GFP-Neo vector. The shRNA sequences for SETD2 or MEC-17 were provided in Supplementary Table 1.

### Animals and genotyping

All animal experiments were approved by Institutional Animal Care and Use Committee, CAS Center for Excellence in Molecular Cell Science, Chinese Academy of Sciences. The C57BL6 mice of either sex at different developmental stages were provided by the Shanghai Laboratory Animal Center of Chinese Academy of Sciences. For genotyping of *Setd2*^*flox/flox*^ and *Mec-17* knockout mice, the genomic DNA was extracted from mouse tail by Proteinase K and amplified by 2 × Taq Plus Master Mix (Vazyme) with indicated primers. The PCR products were analyzed by agarose gel electrophoresis. Genotyping primers were provided in Supplementary Table 2.

### Generation of the antibody against α-TubK40me3

The antibody against α-TubK40me3 was custom-designed and generated (GL Biochem, Shanghai, China). Briefly, the tri-methylated peptide Ac-GQMPSD(KMe3)TIGGGDC-amide conjugated to keyhole limpet hemocyanin was used as the immunogen to generate α-TubK40me3 antibody in rabbits. Purification of α-TubK40me3-specific antibody was performed using serial columns with un-methylated and tri-methylated peptides.

### Cell culture and transfection

HEK293, Neuro-2a and ND7/23 cells (Shanghai cellbank, Chinese Academy of Sciences) were cultured in Dulbecco’s Modified Eagle Medium (DMEM; Gibco) supplemented with 10% fetal bovine serum (Biochrom) and antibiotics (Gibco). The cells were transiently transfected with plasmids using Lipofectamine 2000 (Invitrogen) and then used for different assays after 48 h.

Hippocampal and cortical tissues were dissected from mouse brain at E14 in Hank’s balanced salt solution (HBSS) and digested with 10 U/mL papain (Worthington Bio Corp) and 0.1 mg/mL DNase I (Sigma) for 12 min at 37 °C. After digestion, the tissues were washed in HBSS and then dissociated by repeated passaging through a 1 mL pipette. The indicated plasmids mixed in Nucleofector buffer (0.15 mL) were electroporated into hippocampal and cortical neurons by Amaxa Nucleofector II (Lonza) using program O-005. The neurons were plated on poly-D-lysine (Sigma) coated dishes with Minimum Eagle’s Medium (MEM; Gibco) containing 5% fetal bovine serum. After 3 h, the medium was replaced by Neurobasal medium (Gibco) containing 2% B27 supplement (Gibco) and 2 mM L-Glutamax (Gibco). The neurons were used for immunostaining or time-lapse imaging after 3 days. Fixed neurons which developed one distinct and SMI-312^+^ axon at DIV 3 were defined as the polarized neurons, and all neurites and their protrusions labeled by GFP were counted as neurite tips. For lentivirus infection, the virus at multiplicity of infection (MOI) = 10 was applied to neurons 3 h after plating and removed after 12 h treatment. The percentage of GFP^+^ neurons was observed at DIV 2 and then the neurons were fixed for electron microscopy.

### qPCR

Total mRNAs of mouse cerebral cortex at different development stages were extracted with TRIzol reagent (Invitrogen) and then used for reserve transcription to produce complementary DNAs (cDNAs) by SuperScript II reverse transcriptase (Invitrogen). The primers used to amplify the SETD2, MEC-17 or HDAC6 cDNA were listed at below. For the control, glyceraldehyde-3-phosphate dehydrogenase (GAPDH) cDNA was amplified. The relative mRNA level of individual gene was calculated by a standard curve and normalized to the mRNA level of GAPDH. All values at different stages were normalized to the P60 cerebral cortex. qPCR primers were provided in Supplementary Table 3.

### Protein expression and purification

GST-tagged human SETD2 and its truncated and mutant proteins were expressed in Escherichia coli Rosetta 2 at 37 °C for 4 h, and then induced with 0.5 mM Isopropyl-β-D-Thiogalactoside (Sigma) at 16 °C for 20 h. Cells were collected by centrifugation at 6000 × *g* for 10 min at 4 °C. Cell pellets were resuspended with lysis buffer (50 mM Tris-HCl, pH 8.0, 0.2 mM EDTA, 0.5 mM NaCl, 1 mM PMSF) and then sonicated at 4 °C. Cell fragments were removed by centrifugation at 12,000 × *g* for 40 min at 4 °C and filtered through 0.45 µm filter. The cleaned lysates were absorbed to glutathione sepharose beads (GST-beads; GE Healthcare Biosciences) for 6 h and rinsed with 500 mL wash buffer (50 mM Tris-HCl, pH 8.0, 0.2 mM EDTA) overnight at 4 °C. Proteins were collected in the elution buffer (50 mM Tris-HCl, pH 8.0, 0.2 mM EDTA, 10 mM reduced glutathione), which was replaced by BRB80 (Brinkley Buffer 1980, 80 mM PIPES, 1 mM MgCl_2_, 1 mM EGTA, pH 6.8) later. Protein was frozen by liquid nitrogen and stored in −80 °C.

### Tubulin purification and enzymatic modification

Tubulin was purified from the brain of C57/BL6 mice (8 weeks) by two cycles of polymerization/depolymerization as previously described^39^. The 2 mg tubulin was methylated by adding 0.1 mg purified SETD2(1469-1724) into methylation reaction buffer [50 mM Tris-HCl, pH 8.0, 2 mM MgCl_2_, 0.01% Triton X-100, 1 mM dithiothreitol, 0.5 mM S-(5’-Adenosyl)-L-methionine chloride dihydrochloride (SAM, Sigma)] at 37 °C for 90 min, and then the methylation reaction buffer was replaced by BRB80. The methylated tubulin was frozen by liquid nitrogen and stored in −80 °C.

### Immunoprecipitation and GST pull-down assay

The brain tissues and HEK293 cells were lysed in ice-cold RIPA buffer (50 mM Tris-HCl, PH 7.4, 150 mM NaCl, 1% Triton-X100, 1 mM EDTA, 10% glycerol, 1 mM PMSF, 1 µg/mL aprotinin, 1 µg/mL leupeptin and 1 µg/mL pepstatin). For brain tissues at different developmental stages, the volume of lysis buffer was adjusted to ensure the same input of total α-tubulin. After centrifugation at 15,000 × *g* for 10 min, the supernatant was diluted 10 times and incubated with α-TubK40me3 antibody (1:200; homemade) or H3K36me3 antibody (1:200; Abcam) overnight at 4 °C and then precipitated by protein G agarose (Sigma). For denatured immunoprecipitation, brain tissues were first lysed and boiled in 1% SDS to fully linearize proteins and expose epitopes, and the lysates were diluted 10 times with ice-cold RIPA buffer. Then, immunoprecipitation was performed with α-TubK40me3 antibody overnight at 4 °C and then precipitated by protein G agarose. The immunoprecipitates and 1-2% total lysates were analyzed by immunoblotting.

For GST pull-down assay, purified tubulin from mouse brain was incubated with GST-tagged truncated or mutant SETD2 proteins for 4 h at 4 °C. The protein complex was precipitated by GST-beads and analyzed by immunoblotting.

### Immunoblotting and dot blotting

The protein samples were separated by SDS-PAGE, transferred to the nitrocellulose membrane (Whatman), blocked by 5% skim milk for 60 min at room temperature, probed with specific antibodies and then visualized with enhanced chemiluminescence (Tanon). For dot blotting, indicated peptides were dissolved in PBS and dropped on the nitrocellulose membrane. The primary antibodies include anti-α-tubulin (1:10000; Sigma T5168), α-TubK40ac (1:50000; Sigma T7451), α-TubK40me3 (1:1000; homemade), H3K36me3 (1:10000; Abcam ab9050), SETD2 (1:500; Sigma HPA042451), Tuj1 (1:1000; Abcam ab107216), Flag (1:1000; Sigma F7425/F3165), PH3 (1:1000; Millipore 06-570), β-actin (1:1000; Chemicon MAB1501), GAPDH (1:10000; Abcam ab8245) and GST (1:1000; Abcam ab6612). The blot exposure times were always within the linear range of detection and protein bands were quantified using ImageJ software (NIH).

### Immunostaining

The HEK293 cells or cultured neurons were fixed with 4% paraformaldehyde (PFA) for 20 min at room temperature, blocked by 1% bovine serum albumin (BSA) for 30 min at room temperature, and then incubated with primary antibodies diluted in the buffer containing 1% BSA and 0.3% Triton X-100 against α-tubulin (1:10000; Sigma T5168), SETD2 (1:1000; Sigma HPA042451), α-TubK40me3 (1:1000; homemade), GFP (1:2000; Abcam ab13970), SMI-312 (1:500, Biolegend 837904) or Flag (1:1000; Origene TA50011-100) overnight at 4 °C. The samples were then incubated with corresponding Alexa-Fluor-conjugated secondary antibodies (1:1000; Molecular probe) and DAPI (1:2000; Sigma) for 60 min at room temperature.

The brain tissues of E14, E16 and E18 mice were dissected, post-fixed with 4% PFA for 24 h, and dehydrated in 30% sucrose overnight at 4 °C. The 50 µm-thick sections were cut by a cryostat and incubated with primary antibodies diluted in the buffer containing 1% BSA and 0.3% Triton X-100 against Tuj1 (1:1000; Abcam ab107216), α-TubK40me3 (1:1000; homemade), SETD2 (1:1000; Sigma HPA042451), Ki67 (1:500; BD Biosciences 550609), PH3 (1:1000; Millipore 06-570), cleaved caspase 3 (1:1000; CST 9661S), Nestin (1:1000, Millipore MAB353) or Flag (1:1000; Origene TA50011-100) for 24 h at 4 °C. Notably, a permeabilization and blocking step before primary antibody with 5% BSA and 1% Triton X-100 for 2 h at 4 °C was benefit for α-TubK40me3 and H3K36me3 staining in the brain sections. The sections were then incubated with corresponding Alexa-Fluor-conjugated secondary antibodies (1:1000; Molecular Probe) and DAPI (1:2000; Sigma) for 2 h at room temperature and mounted on gelatin-coated slides. All images of immunostaining were collected with a TCS SP8 confocal microscope (Leica).

For quantitative analysis of SETD2 expression, α-TubK40me3 and H3K36me3 level in brain slices, the mean fluorescence intensities of SETD2, α-TubK40me3 and H3K36me3 staining within GFP^+^ neurons were measured and normalized to those fluorescence intensities of neighboring GFP-negative (GFP^-^) neurons.

### In utero electroporation

The uteruses of mice at gestation day 14 were exposed, and 3 µg of indicated plasmids mixed with 0.75 µg GFP plasmid and 0.1 µL of Fast Green (2 mg/mL; Sigma) were injected into lateral ventricle of the embryo. Next, electric pulses were generated by an ElectroSquireportator T830 (BTX) and applied to the cerebral wall for 5 repetitions of 30 V for 50 ms with an interval of 1 s. The uterine horns were then replaced in the abdominal cavity, and the abdominal wall and skin were sutured using a surgical needle and thread. At the indicated time point, the mouse brains were dissected and processed for immunostaining.

For analysis of neuronal position in the cortical wall, three to ten puppy brains (select two sections for each brain) from at least three individual mother mice were used for each experimental condition with ~500 GFP^+^ cells counted in each section. The somatosensory cortex of embryonic brain was imaged for analyzing radial migration of cortical neurons. The different subregions of cortical wall were identified based on cell density after staining with DAPI. For analysis of neuronal morphology in the IZ, 20-100 GFP^+^ cells were selected from each brain section. For analysis of the percentage of Ki67/PH3/cleaved caspase 3-positive cells in the VZ/SVZ, 100-300 GFP^+^ cells were selected from each brain section. All quantitative data were collected from at least 3 brains under each experimental condition and the sample size (n) in the figure legends represented the number of brain sections.

### Measurement of the soluble and polymerized tubulin fractions

HEK293 cells and neurons were lysed with pre-warmed BRB80 (Brinkley Buffer 1980, 80 mM PIPES, 1 mM MgCl_2_, 1 mM EGTA, pH 6.8) supplemented with 0.5% Triton X-100 and 1 μM Taxol for 5 min and centrifuged at 17,400 × *g* for 15 min at room temperature. The supernatant was denatured with loading buffer and used as soluble tubulin fraction (S). The pellet was resuspended with cold BRB80 buffer supplemented with 1% Triton X-100 and placed on ice for 15 min to depolymerize microtubules. After centrifugation at 15,000 × *g* for 10 min, the supernatant was denatured and used as the polymerized microtubule fraction (P). Then, the volume of S and P fractions were adjusted to equal and analyzed by immunoprecipitation and immunoblotting.

### Sedimentation assay

To remove aggregated tubulin and oligomers, free tubulin solution was centrifuged at 126,000 × *g* for 10 min at 4 °C. Then, 10 µM tubulin supernatants were incubated in BRB80 for 30 min at 37 °C mixed with 20 μM Taxol, 1 mM GTP and 5% glycerol. Supernatant and pellet were collected after centrifugation at 150,000 × *g* for 25 min at 27 °C, and the pellet was resuspended with equal volume sample buffer. Proteins in each fraction were analyzed by immunoblotting.

### In vitro microtubule reconstitution assay

In order to generate rhodamine- or HiLyte-647-labeled GMPCPP-stabilized microtubule seeds, unlabeled mouse brain tubulin dimers, biotin-tubulin (Cytoskeleton) and rhodamine-tubulin (Cytoskeleton) or HiLyte-647 tubulin (Cytoskeleton) were mixed at ratio of 8:1:1 and incubated with 1 mM GMPCPP (Jena Biosciences) at 37 °C for 30 min. The mixture was then centrifuged at 126,000 × *g* for 5 min at 27 °C to remove unpolymerized tubulin. Cold BRB80 was added to resuspend the microtubule pellet, followed by depolymerization at 4 °C for 20 min and a second round of polymerization with 1 mM GMPCPP at 37 °C for 30 min. Finally, microtubule seeds were obtained after centrifugation at 126,000 × *g* for 5 min at 27 °C, resuspended using 37 °C pre-warmed BRB80, frozen by liquid nitrogen and stored in −80 °C.

For the assembly of sample chamber, cleaned and silanized glass coverslip was attached onto microscopic slides by double-sided tape to create a reacting space. Low or high level of methylated tubulin and HiLyte-488 labeled tubulin (at a 19:1 ratio) were mixed with reaction buffer containing 5 mM Dithiothreitol (DTT), 0.1% methylcellulose, 75 mM KCl, 0.25 mg/mL bovine serum albumin (BSA), 25 mM glucose, 100 µM catalase, 300 µM glucose oxidase and 1 mM GTP. For microtubule growth assay, microtubule seeds were flowed through the channel for 10 min at 37 °C before adding reaction buffer.

For image collection, sealed chamber was put into Zeiss cell observer spinning disk system with a pre-warmed 37 °C temperature-controlled workstation. Images were acquired with a 100 × oil objective (NA = 1.40) and an Orca CCD camera (Hamamatsu). Tubulin nucleation and growing microtubules from seeds were imaged by TIRF every 5 s for 30 min and every 3 s for 15 min, respectively. ImageJ software (NIH) and Image-Pro Plus 5 software (Media Cybernetics) were used to generate kymographs and further analyzed respectively.

### Time-lapse imaging

To detect the movement of EB3 tagged with tdTomato (EB3-tdTomato), E14 hippocampal neurons were transfected with indicated plasmids and then plated on glass-bottom dishes. At DIV 3, the dishes were placed on a temperature-controlled workstation (37 °C, 5% CO_2_) with an inverted microscope. Randomly selected neurons (at least 35 neurons for each condition from 4 individual experiments) were imaged using the Zeiss cell observer spinning disk system (Zeiss) with Zen 2.1 software under 60 × oil lens and 60 frames were collected for 2 min at 2-s interval. The number of EB3 comets in the cell body was averaged from 3 independent frames. The neurite segments were selected from the middle region of the longest neurite in neurons. The kymograph was generated using ImageJ software (NIH), and then the number and direction of EB3 comets in the neurite were analyzed. The mean velocity, growth lifetime, and catastrophe frequency of EB3 comets in particular kymograph was calculated using Image-Pro Plus 5.1 software.

### Proximity ligation assay

The proximity ligation assay was performed according to the Duolink manufacturer’s instructions (Sigma). In brief, E14 hippocampal neurons grown on glass slides at DIV 3 were fixed with 4% PFA for 20 min at room temperature, blocked by 1% BSA for 30 min at room temperature and incubated with indicated primary antibodies diluted in the buffer containing 1% BSA and 0.3% Triton X-100 overnight at 4 °C. Then, neurons were incubated with secondary antibodies conjugated with oligonucleotides at 37 °C for 1 h and performed with the ligase in the ligation solution for 30 min at 37 °C. After rolling-circle amplification using the polymerase for 100 min at 37 °C in the dark, final immunostaining was processed using sheep antibody against tubulin (1:500; Cytoskeleton ATN02) or rat antibody against tyrosinated tubulin (1:1000; Abcam ab6160).

### Electron microscopy

For transmission electron microscopy, cultured E14 hippocampal neurons on glass-bottom dishes were fixed in 2.5% glutaraldehyde overnight at 4 °C. Cells were then post-fixed with 1% OsO_4_ for 30 min at room temperature. The samples were dehydrated with graded ethanol series and embedded in Epon 812 resin. The cells were removed from dishes and re-embedded on edge to ensure cross-section of neurites. The 70 nm ultrathin sections were stained with 2% uranyl acetate for 10 min and 1% lead citrate for 5 min. Images were captured at 80 kV using a Tecnai G2 Spirit transmission electron microscopy (FEI).

### Statistical analysis

All data were collected from at least 3 independent experiments and presented as the mean ± s.e.m. No statistical methods were used to predetermine the sample sizes. Statistical analysis was performed using Prism 5 software (GraphPad). For Western blot and in vitro microtubule reconstitution assay, data were analyzed using paired Student’s *t* test. In other experiments, comparison between two groups was performed with unpaired Student’s *t* test and comparison among multiple groups was evaluated by two-way analysis of variance (ANOVA) with Bonferroni’s post-hoc test. Differences were considered significant at the level of *P* < 0.05.

### Reporting summary

Further information on research design is available in the Nature Research Reporting Summary linked to this article.

## Supplementary information

Supplementary Information

Reporting Summary

## Data Availability

The authors declare that the data supporting the findings of this study are available within the paper and its supplementary information files. Data are available from the corresponding author upon reasonable request. Source data are provided with this paper.

## Source data

Source Data
