# Supplementary material for: α-TubK40me3 is required for neuronal polarization and migration by promoting microtubule formation
Source: Nat Commun. 2021 Jul 5;12:4113. doi: 10.1038/s41467-021-24376-2 (PMC8257576; doi:10.1038/s41467-021-24376-2)
Supplement: Supplementary file 1 — Supplementary Information [file 41467_2021_24376_MOESM1_ESM.pdf]

Supplementary Table 1 shRNA sequences

mSETD2 shRNA1	5’-ATAGTGTGACCTCGCCTTATT-3’
mSETD2 shRNA2	5’-ACTTTGTGAGGATAGTATAAA-3’
MEC-17 shRNA	5’-GCAGCAAATCATGACTATTGT-3’

Supplementary Table 2 Primers for genotyping

Setd2-F	5’-CACAGTATTCCAGGACAAAGGTGT-3’
Setd2-R	5’-TATTTAAACTCTCTCTGGGGGTGG-3’
Mec-17-F	5’-GGCTGCCAGGAATGACTTACACG-3’
Mec-17-R	5’-CAGGGAATAATGACAGTAAGACTCACG-3’
Mec-17-NeoF	5’-GCAGCCTCTGTTCCACATACACTTCA-3’
Mec-17-NeoR	5’-TAGACTGTTTCCTGGGTTCTACTGCC-3’

Supplementary Table 3 Primers for qPCR

SETD2-F	5’-CCCAGCAACAGCACTAATGAC-3’
SETD2-R	5’-TCCACCTCTAAAACTTCGGGTC-3’
MEC-17-F	5’-TGTACTGGATGACCGGGAGG-3’
MEC-17-R	5’-GCTGAAAAAGTTCTCGCCCG-3’
HDAC6-F	5’-TCCACCGGCCAAGATTCTTCT-3’
HDAC6-R	5’-CAGCACACTTCTTTCCACCAC-3’
GAPDH-F	5’-TATGTCGTGGAGTCTACTGGTGTCTTCACC-3’
GAPDH-R	5’-GTTGTCATATTTCTCGTGGTTCACACCC-3’

Extended Data Figures

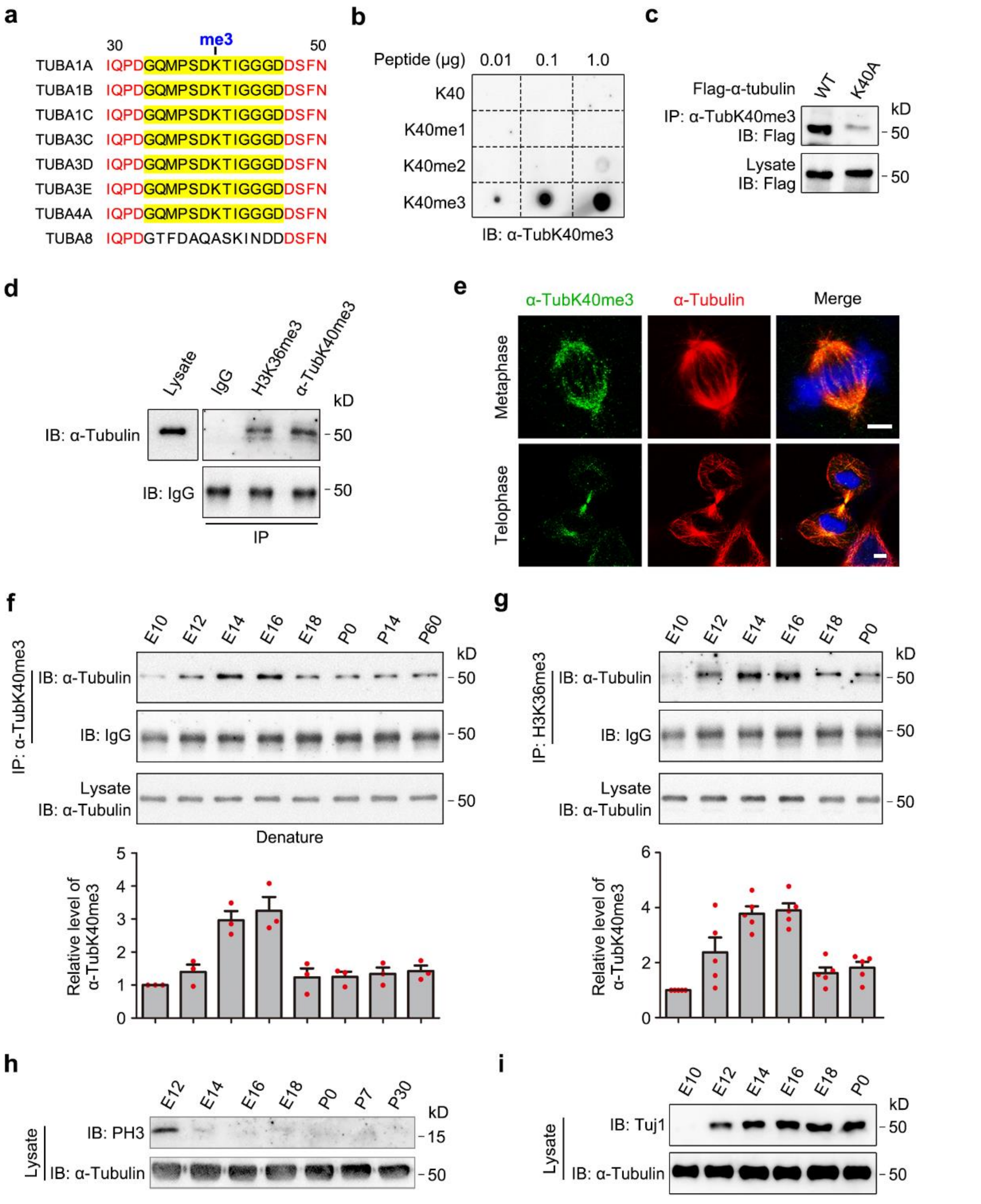

Extended Data Figure 1 Validation of homemade α-TubK40me3 antibody and detection of α-TubK40me3, Tuj1 and PH3 during neuronal development

(a) Alignment of the sequence in 30-50 amino acids of different human α-tubulin isotypes. The immunogen peptide containing tri-methylated lysine 40 is highlighted in yellow, which is highly conserved in most α-tubulin isotypes. (b) Unmodified (K40),

mono-methylated (K40me1), di-methylated (K40me2) and tri-methylated (K40me3) peptide of α-tubulin (0.01 μg, 0.1 μg and 1.0 μg) were dot-blotted on nitrocellulose membrane, and our homemade α-TubK40me3 antibody specifically recognized the tri-methylated peptide (n = 3 biological replicates). (c) Immunoprecipitation by α-TubK40me3 antibody and following immunoblotting with Flag antibody in HEK293 cells expressing Flag-α-tubulin or Flag-α-tubulin^{K40A} showed that our homemade α-TubK40me3 did not recognize methylation-deficient Flag-α-tubulin^{K40A} (n = 3 biological replicates). (d) Immunoprecipitation by α-TubK40me3 or H3K36me3 antibody and following immunoblotting with α-tubulin in E16 brain lysate indicated that both antibodies were able to detect methylated α-tubulin (n = 3 biological replicates). (e) Immunostaining by α-TubK40me3 (green) and α-tubulin (red) antibodies in HEK293 cells showed that α-TubK40me3 was mainly distributed in the spindle and the midbody. Cells were stained for DAPI (blue) (n = 3 biological replicates). Scale bar: 5 μm. (f) Denature immunoprecipitation by α-TubK40me3 antibody and following immunoblotting with α-tubulin showed the temporal regulation of α-TubK40me3 at different developing stages of mouse cerebral cortex. α-Tubulin and IgG served as the loading control of whole protein and antibody, respectively. The data were normalized to E10 and shown as the mean ± s.e.m. (n = 3 biological replicates). (g) Immunoprecipitation by H3K36me3 antibody and following immunoblotting with α-tubulin showed the temporal regulation of α-TubK40me3 at different developing stages of mouse embryonic cerebral cortex. α-Tubulin and IgG served as the loading control of whole protein and antibody, respectively. The data were

normalized to E10 and shown as the mean ± s.e.m. (n = 5 biological replicates). (h) Immunoblotting of PH3 in cerebral cortex from embryonic to adult stage showed the high level at E12 (n = 3 biological replicates). (i) Immunoblotting of neuronal marker Tju1 in embryonic cerebral cortex showed abundant neurogenesis after E12 (n = 3 biological replicates).

Source data are provided as a Source Data file.

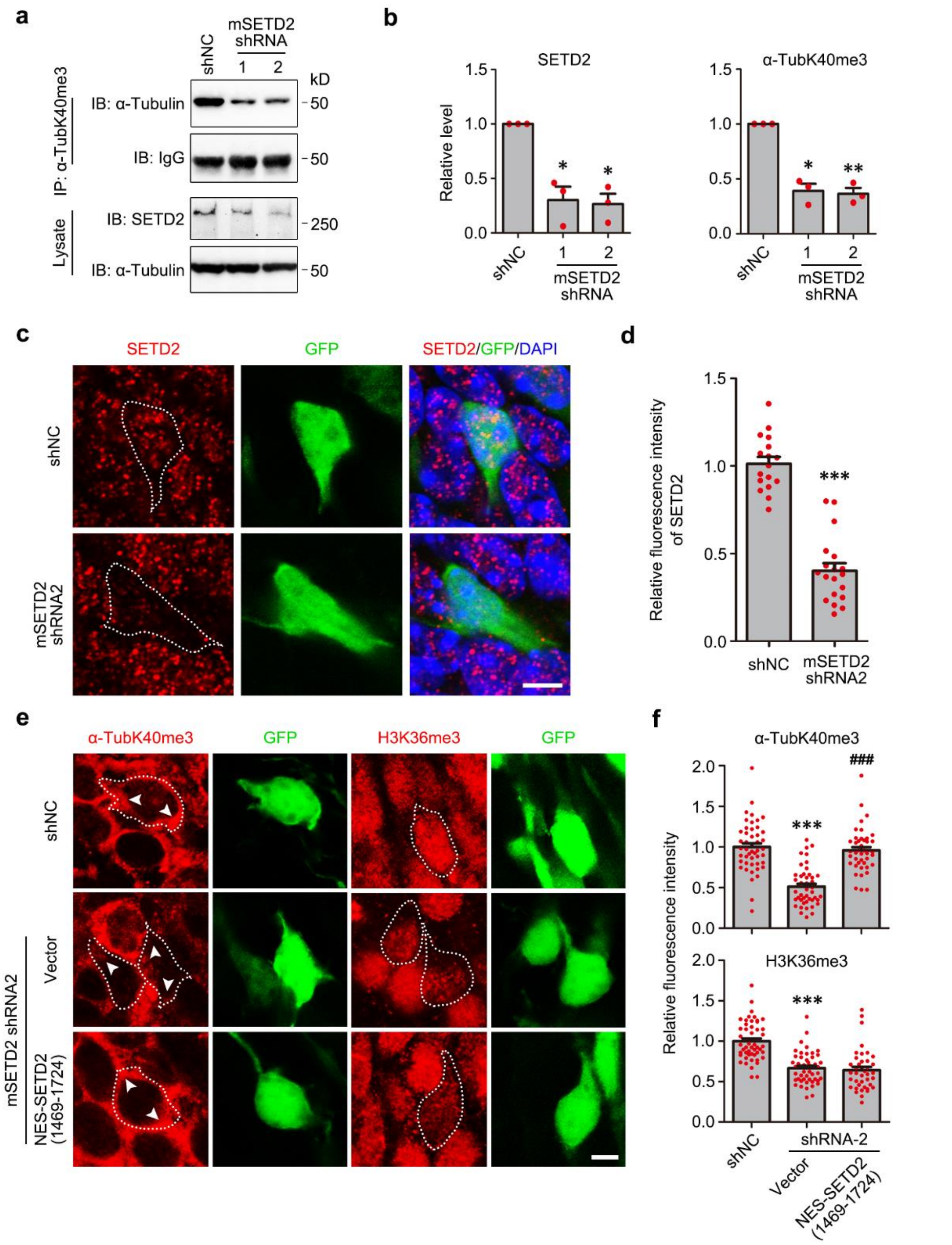

Extended Data Figure 2 The knockdown efficiency of SETD2 shRNA and the rescue effects of NES-SETD2(1469-1724)

(a) Immunoprecipitation and immunoblotting showed the levels of SETD2 and α-TubK40me3 in Neuro-2a cells transfected with mSETD2 shRNAs for 48 h. α-Tubulin and IgG served as loading control of whole protein and antibody, respectively. (b)

Quantitative analysis of (a) showed that the level of SETD2 and α-TubK40me3 was highly reduced after transfection with mSETD2 shRNAs. All data were normalized to shNC and shown as the mean ± s.e.m. (n = 3 biological replicates). Data were analyzed using paired two-tailed Student's *t*-test and $^{*}P < 0.05$, $^{**}P < 0.01$ versus shNC. (c) Representative images of SETD2 puncta (red) in shNC and mSETD2 shRNA2-expressing neurons (green) at E16 in brain sections. Brain slices were stained for DAPI (blue). Scale bar: 5 μm. (d) Quantitative analysis of (c) showed that the intensity of SETD2 staining was largely decreased in mSETD2 shRNA2-expressing neurons. All data were shown as the mean ± s.e.m. (17 and 19 neurons from 3 brains respectively). Data were analyzed using unpaired two-tailed Student's *t*-test and $^{***}P < 0.001$ versus shNC. (e) Representative images of α-TubK40me3 or H3K36me3 staining (red) in GFP^{+} cells (green) electroporated with GFP reporter as well as shNC, mSETD2 shRNA2 or mSETD2 shRNA2 together with NES-SETD2(1469-1724) in E16 brain sections. Scale bar: 5 μm. (f) Quantitative analysis of fluorescence intensity (normalized to neighbouring GFP^{-} neurons) from (e) showed that both the level of α-TubK40me3 and H3K36me3 were decreased after SETD2 knockdown in E16 brain sections, but NES-SETD2(1469-1724) only restored the level of α-TubK40me3. All data were shown as the mean ± s.e.m. (47, 45 and 42 neurons from 3 brains respectively for α-TubK40me3; 52, 50 and 40 neurons from 3 brains respectively for H3K36me3). Data were analyzed using unpaired two-tailed Student's *t*-test and $^{***}P < 0.001$ versus shNC; $^{###}P < 0.001$ versus shRNA2.

Source data are provided as a Source Data file.

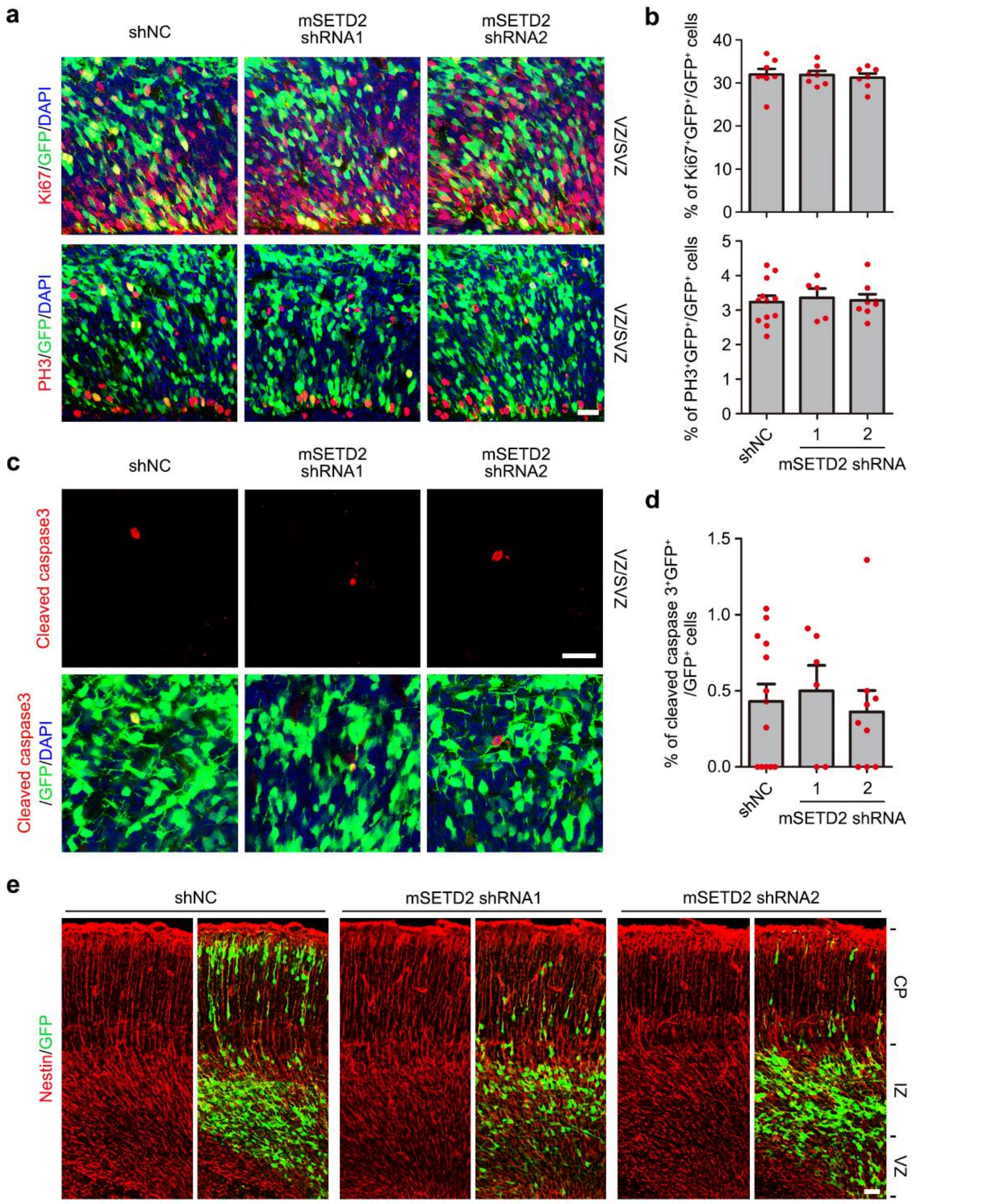

Extended Data Figure 3 SETD2 knockdown does not alter the proliferation, survival or basal processes of NPCs

(a and c) Representative images of coronal brain sections at E16 showed the proliferative, mitotic and apoptotic cells in VZ/SVZ region, which were accessed by staining the Ki67 (red), PH3 (red) and cleaved caspase3 (red), respectively, after electroporated with GFP reporter as well as shNC, SETD2 shRNA1 or SETD2 shRNA2

at E14. Sections were stained for DAPI (blue). Scale bar: 25 μm. (b and d) Quantitative analysis of (a) and (c) showed that the percentage of $Ki67^+$ (n = 8, 7, 7, respectively), $PH3^+$ (n = 12, 5, 8, respectively) or cleaved caspase 3^+ (n = 13, 6, 9, respectively) cells was not changed after SETD2 knockdown in all GFP^+ cells. All data were shown as the mean ± s.e.m. Data were analyzed using unpaired two-tailed Student's *t*-test. (e) Immunostaining showed that the basal processes of radial glial progenitor projected properly to serve as the guide for migrating neurons in both control and SETD2 knockdown brains at E16. Sections were stained for Nestin (red) and DAPI (blue) (n = 3 biological replicates). Scale bar: 25 μm.

Source data are provided as a Source Data file.

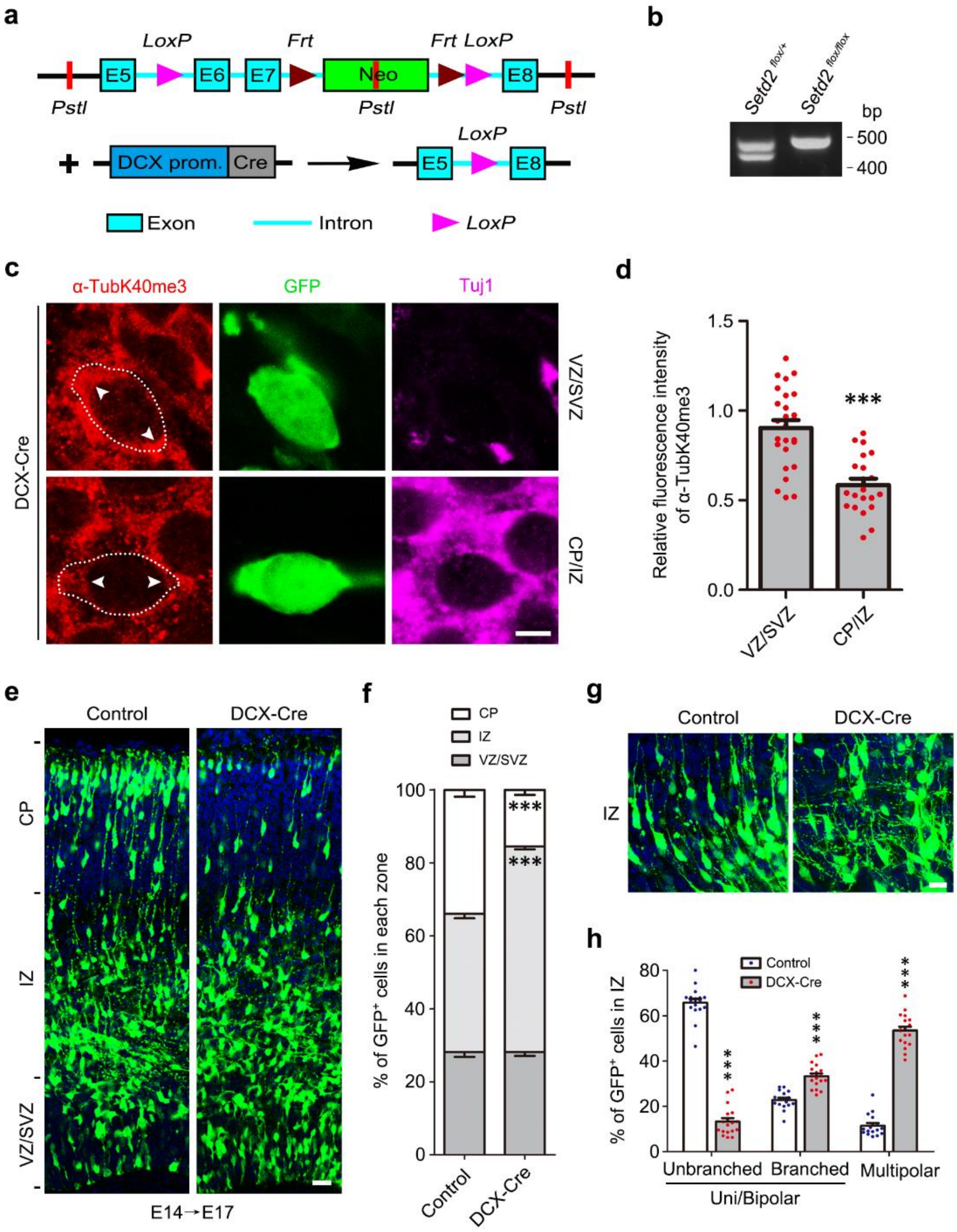

Extended Data Figure 4 Neuron-specific deletion of SETD2 results in defects of neuronal morphology transition and migration

(a) The schematic of *Setd2* conditional knockout strategy. The exon 6-7 of SETD2-coding gene was flanked by LoxP sites and deleted by doublecortin promoter driven Cre (DCX-Cre) in neurons. (b) Representative genotyping results. The length of genotyping product for Flox allele with *LoxP* sites was longer than the WT (+) allele. (c) Representative images of α-TubK40me3 staining (red) in GFP^{+} cells (green)

electroporated with GFP reporter as well as DCX-Cre in VZ/SVZ and CP/IZ regions of E17 brain sections from *Setd2*$^{flox/flox}$ mice. Brain slices were stained for Tuj1 (magenta). Scale bar: 5 μm. (d) Quantitative analysis of fluorescence intensity (normalized to neighbouring GFP^{-} neurons) from (c) showed that the level of α-TubK40me3 in CP/IZ but not VZ/SVZ were decreased after DCX-Cre transfection. All data were shown as the mean ± s.e.m. (25 and 20 neurons from 3 brains respectively). Data were analyzed using unpaired two-tailed Student's *t*-test and ***P < 0.001 versus VZ/SVZ. (e) Representative images of somatosensory cortex from *Setd2*$^{flox/flox}$ mice at E17 showed the distribution of GFP^{+} cells (green) electroporated with GFP reporter as well as DCX-Cre at E14. Sections were stained for DAPI (blue). Scale bar: 25 μm. (f) Quantitative analysis of (e) showed that the percentage of GFP^{+} cells in IZ was significantly increased after DCX-Cre transfection. All data were shown as the mean ± s.e.m. (n = 18, 18, respectively). Data were analyzed using two-way ANOVA with Bonferroni's post-hoc test and ***P < 0.001 versus control. (g) Representative images of control and DCX-Cre-expressing neurons (green) at E17 in the IZ. Brain slices were stained for DAPI (blue). Scale bar: 10 μm. (h) Quantitative analysis of (g) showed that the percentage of GFP^{+} neurons at multipolar stage in IZ was significantly increased after DCX-Cre transfection. All data were shown as the mean ± s.e.m. (n = 17, 18, respectively). Data were analyzed using two-way ANOVA with Bonferroni's post-hoc test and ***P < 0.001 versus control.

Source data are provided as a Source Data file.

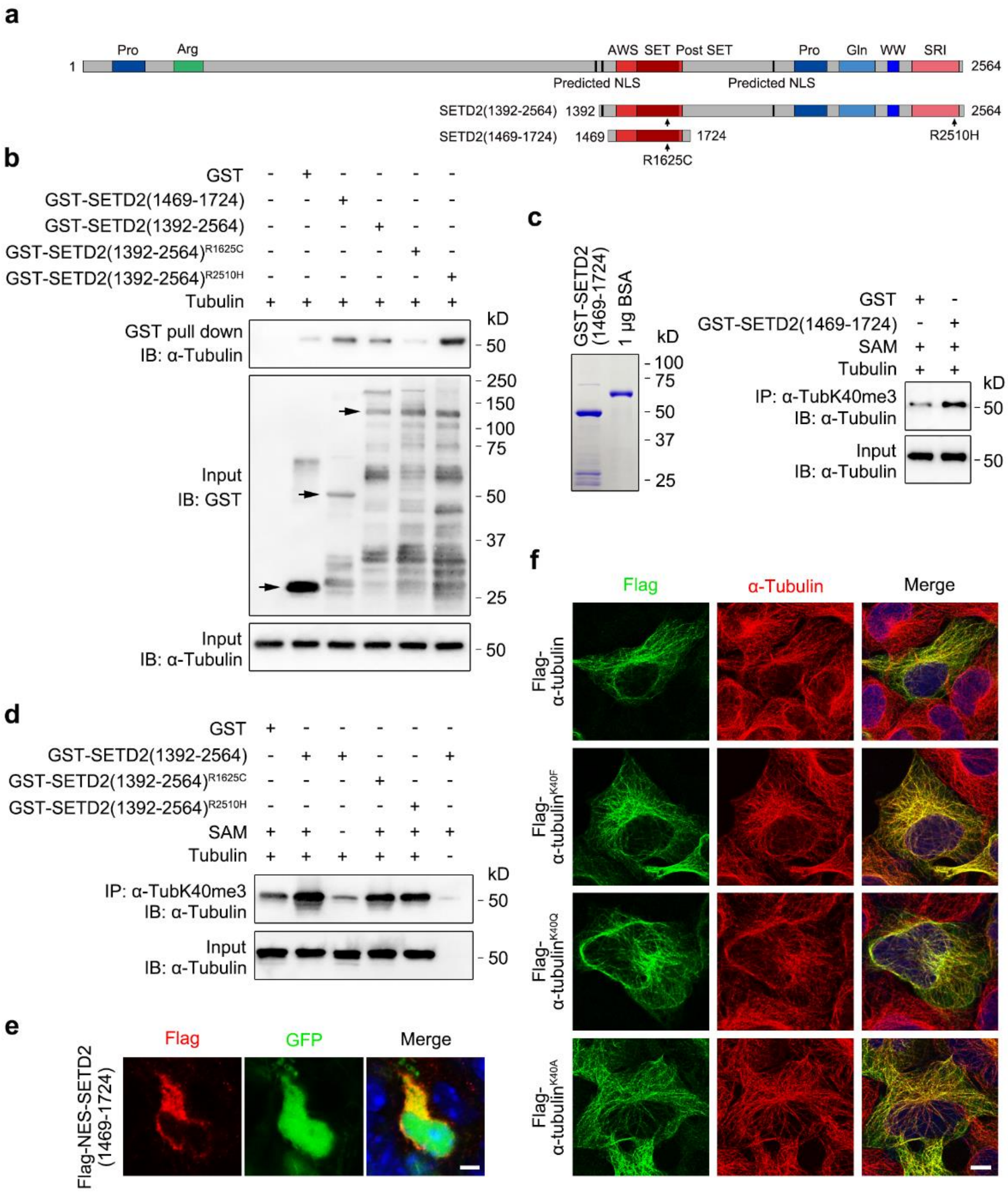

Extended Data Figure 5 ***In vitro*** **binding and catalytic activity of various truncations and mutants of SETD2 and incorporation ability of α-tubulin K40 mutants**

(a) Schematic for the functional domains within full-length mouse SETD2 and various truncations as well as mutants. (b) GST pull-down of purified tubulin with GST-fused SETD2(1469-1724), SETD2(1392-2564), SETD2(1392-2564)^{R1625C} or SETD2(1392-2564)^{R2510H} showed that all SETD2 truncations and mutants except SETD2(1392-

2564)^{R1625C} could interact with α-tubulin. GST and α-tubulin were immunoblotted by respective antibody. Arrowheads indicate the band of GST and GST-fused proteins (n = 2 biological replicates). (c) Coomassie blue staining and catalytic activity of purified GST-SETD2(1469-1724) (n = 3 biological replicates). Bovine serum albumin (BSA) was introduced for relative quantification. (d) *In vitro* methylation of GST-fused SETD2(1392-2564), SETD2(1392-2564)^{R1625C} and SETD2(1392-2564)^{R2510H} showed that all SETD2 truncations and mutants displayed methyltransferase activity towards α-tubulin (n = 3 biological replicates). (e) Immunostaining of Flag (red) and DAPI (blue) in brain sections showed that Flag-NES-SETD2(1469-1724) was localized in the cytoplasm of GFP^{+} cells (green) (n = 3 biological replicates). Scale bar: 5 μm. (f) Immunostaining of Flag (green) and α-tubulin (red) in HEK293 cells showed that Flag-tagged α-tubulin and various mutants could be well incorporated into microtubule networks. Cells were stained for DAPI (blue) (n = 3 biological replicates). Scale bar: 5 μm.

Source data are provided as a Source Data file.

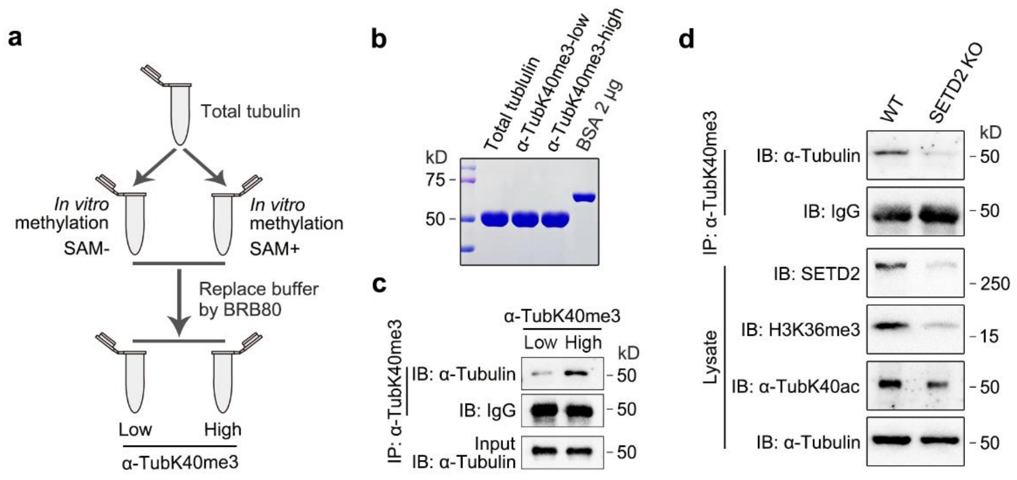

Extended Data Figure 6 *In vitro* preparation of highly methylated α-tubulin and validation of SETD2 knockout cells

(a) Work flow to obtain low and high methylation level tubulins. (b) Coomassie blue staining of total tubulin and tubulins with low and high methylation level after *in vitro* reaction (n = 2 biological replicates). BSA was shown for relative quantification. (c) Immunoprecipitation by α-TubK40me3 antibody and following immunoblotting with α-tubulin showed that the level of α-TubK40me3 was significantly increased after *in vitro* methylation reaction (n = 3 biological replicates). (d) Immunoprecipitation and immunoblotting showed that SETD2 expression, level of α-TubK40me3, H3K36me3 and α-TubK40ac were decreased in SETD2 knockout HEK293 cells (n = 3 biological replicates).

Source data are provided as a Source Data file.

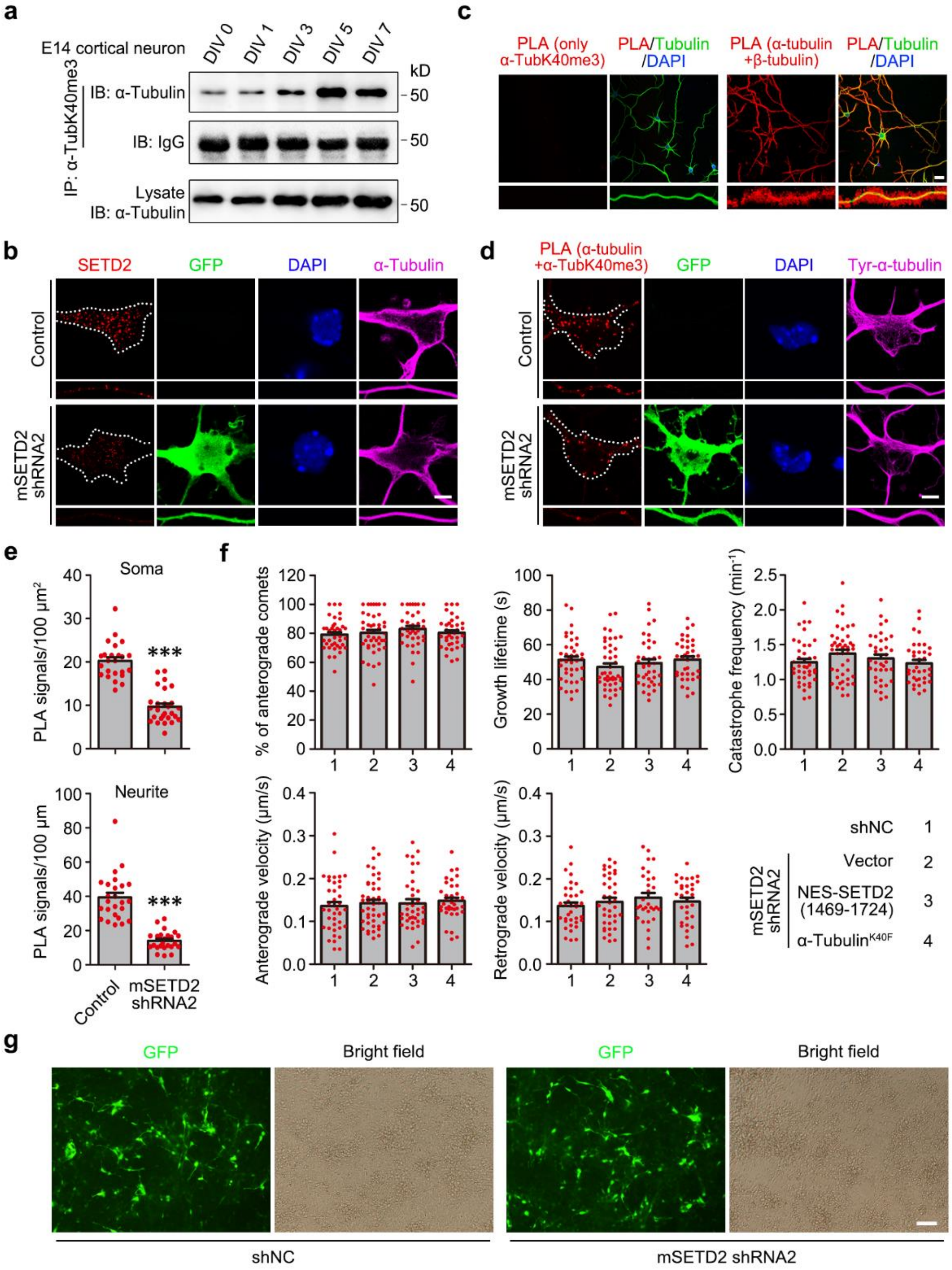

Extended Data Figure 7 Detection of SETD2 localization, level of α-TubK40me3, moving velocity of EB3 comets and lentivirus infection in cultured neurons

(a) Immunoprecipitation by α-TubK40me3 antibody and following immunoblotting with α-tubulin showed that the level of α-TubK40me3 was increased during development of cultured cortical neurons (n = 3 biological replicates). (b)

Immunostaining showed that SETD2 puncta (red) was distributed both in the cell body and neurites of cultured cortical neurons at DIV 3, and significantly decreased after SETD2 knockdown (GFP^+ neurons, green). Neurons were stained for α-tubulin (magenta) and DAPI (blue). Dashed line indicated the lineament of cell (n = 4 biological replicates). Scale bar: 5 μm. (c) PLA of only α-TubK40me3 antibody and α-tubulin/β-tubulin was regarded as the negative and positive control, respectively. Neurons were stained for tubulin (green) and DAPI (blue) after PLA reaction (n = 3 biological replicates). Scale bar: 25 μm. (d and e) Representative images and quantitative analysis showed that PLA signals representing α-TubK40me3 (red) were distributed both in the cell body and neurites of cultured cortical neurons at DIV 3, and significantly decreased after SETD2 knockdown (GFP^+ neurons, green). Neurons were stained for Tyr-α-tubulin (magenta) and DAPI (blue). Dashed line indicated the lineament of cell. Data were analyzed using unpaired two-tailed Student's *t*-test and ***P < 0.001 versus control (23 and 26 neurons respectively for soma; 24 and 23 neurons respectively for neurite). Scale bar: 5 μm. (f) Quantitative analysis showed that the orientation, growth lifetime, catastrophe frequency and velocity of EB3 comets in the neurites were not significantly changed after SETD2 knockdown or together with expression of NES-SETD2(1469-1724) and α-$tubulin^{K40F}$. All data were shown as the mean ± s.e.m. (41, 44, 39 and 35 neurons respectively collected from 3 biological replicates with similar results). Data were analyzed using unpaired two-tailed Student's *t*-test. (g) Representative images of cultured neurons at DIV 2 showed that most of neurons were GFP-positive and infected with lentivirus (n = 2 biological replicates). Scale bar: 30

μm.

Source data are provided as a Source Data file.

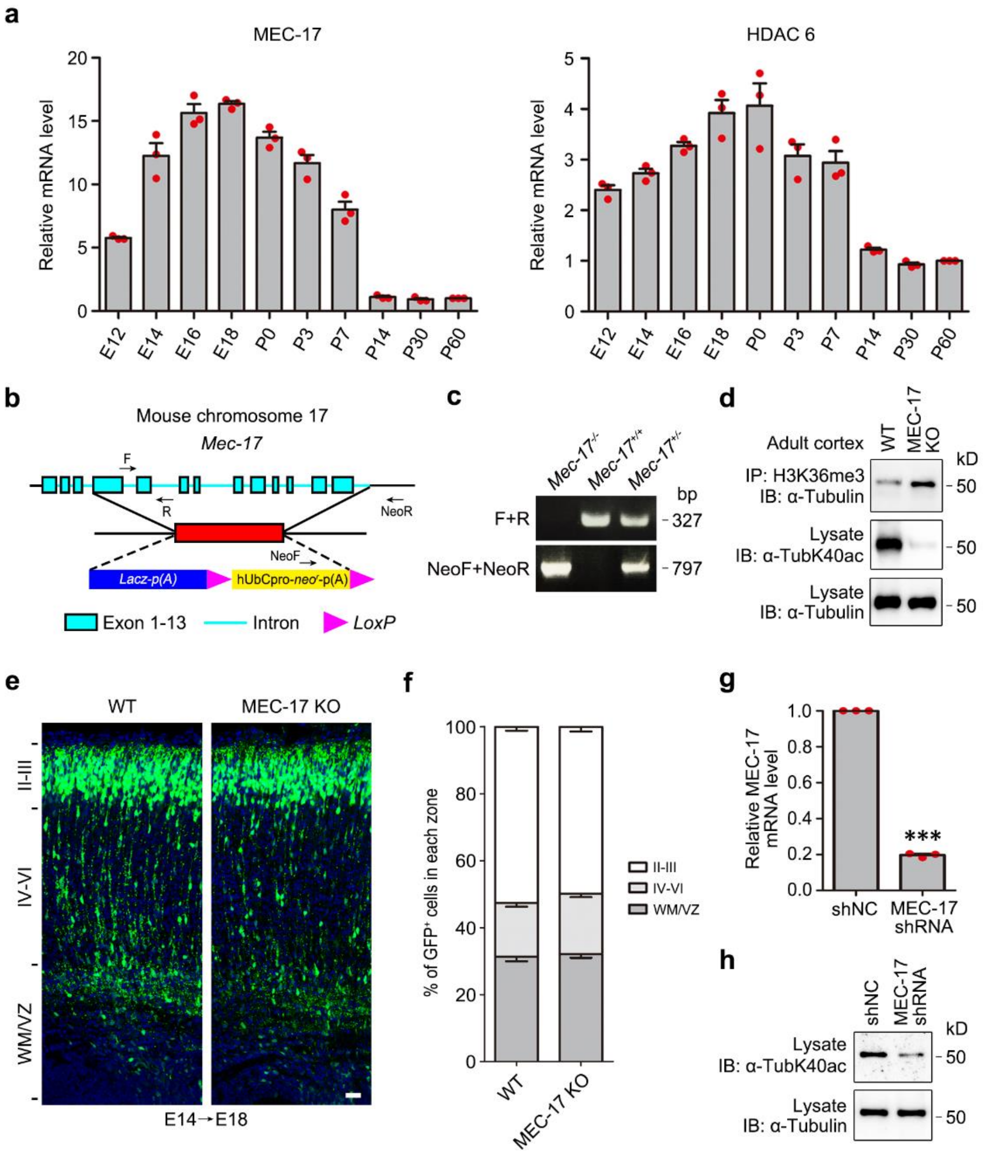

Extended Data Figure 8 Crosstalk between α-TubK40me3 and α-TubK40ac, neuronal migration in MEC-17 knockout mice and validation of MEC-17 shRNA

(a) qPCR showed that the relative level of MEC-17 and HDAC6 mRNA was high in late embryonic and early post-natal mouse cerebral cortex. All data were normalized to the P60 cerebral cortex and represented as the mean ± s.e.m. (n = 3 biological replicates).

(b) The schematic of *Mec-17* knockout strategy. The exon 4-13 of MEC-17-coding gene was deleted by homologous recombination. Two pairs of primers were designed to detect the WT (F and R) and knockout allele (NeoF and NeoR) respectively. (c) Representative genotyping results. The length of genotyping product was 327 base pairs (bp) for the WT allele and 797 bp for the MEC-17 knockout allele. (d) Immunoprecipitation by H3K36me3 antibody and following immunoblotting with α-tubulin showed that the level of α-TubK40me3 was increased in adult cortex of MEC-17 knockout mice (n = 4 biological replicates). (e) Representative images of coronal brain sections from WT and MEC-17 knockout mice in somatosensory cortex at E18 showing the distribution of GFP^+ cells (green) electroporated with GFP reporter at E14. Sections were stained for DAPI (blue). Scale bar: 30 μm. (f) Quantitative analysis of (e) showed that the percentage of GFP^+ cells in different regions was similar between WT and MEC-17 knockout mice. All data were shown as the mean ± s.e.m. (n = 10, 14, respectively). (g) qPCR showed that the level of MEC-17 mRNA was significantly decreased in ND7/23 cells transfected with MEC-17 shRNA for 48 h. Data were normalized to shNC and represented as the mean ± s.e.m. (n = 3 biological replicates). Data were analyzed using paired two-tailed Student's *t*-test and ***$P < 0.001$ versus shNC. (h) Immunoblotting showed that the level of α-TubK40ac was reduced in ND7/23 cells transfected with MEC-17 shRNA for 48 h (n = 3 biological replicates).

Source data are provided as a Source Data file.
