# Supplementary material for: α-TubK40me3 is required for neuronal polarization and migration by promoting microtubule formation
Source: Nat Commun. 2021 Jul 5;12:4113. doi: 10.1038/s41467-021-24376-2 (PMC8257576; doi:10.1038/s41467-021-24376-2)
Supplement: Supplementary file 2 — Reporting Summary [file 41467_2021_24376_MOESM2_ESM.pdf]

nature research

Corresponding author(s): Lan Bao, Wenfeng Feng

Last updated by author(s): May 6, 2021

Reporting Summary

Nature Research wishes to improve the reproducibility of the work that we publish. This form provides structure for consistency and transparency in reporting. For further information on Nature Research policies, see our Editorial Policies and the Editorial Policy Checklist.

Statistics

For all statistical analyses, confirm that the following items are present in the figure legend, table legend, main text, or Methods section.

n/a	Confirmed	
☐	☒	The exact sample size (n) for each experimental group/condition, given as a discrete number and unit of measurement
☐	☒	A statement on whether measurements were taken from distinct samples or whether the same sample was measured repeatedly
☐	☒	The statistical test(s) used AND whether they are one- or two-sided<br>*Only common tests should be described solely by name; describe more complex techniques in the Methods section.*
☐	☒	A description of all covariates tested
☐	☒	A description of any assumptions or corrections, such as tests of normality and adjustment for multiple comparisons
☐	☒	A full description of the statistical parameters including central tendency (e.g. means) or other basic estimates (e.g. regression coefficient) AND variation (e.g. standard deviation) or associated estimates of uncertainty (e.g. confidence intervals)
☐	☒	For null hypothesis testing, the test statistic (e.g. F, t, r) with confidence intervals, effect sizes, degrees of freedom and P value noted<br>*Give P values as exact values whenever suitable.*
☒	☐	For Bayesian analysis, information on the choice of priors and Markov chain Monte Carlo settings
☒	☐	For hierarchical and complex designs, identification of the appropriate level for tests and full reporting of outcomes
☒	☐	Estimates of effect sizes (e.g. Cohen's d, Pearson's r), indicating how they were calculated

Our web collection on statistics for biologists contains articles on many of the points above.

Software and code

Policy information about availability of computer code

Data collection	LAS X (Version 3.1.5, Leica) and Zen 2.1 (Version 11.0, Zeiss) were used for generating fluorescence image data. FEI Tecnai G2 Spirit TEM was used for generating electron microscopy image data. Gel Cap software (Tanon) with ECL method was used for generating Western blot data.
Data analysis	Image-Pro Plus 5.1 software (Media Cybernetics), ImageJ software (NIH, version 1.52i) and its plugins were used for fluorescence image analysis. ImageJ software (NIH, version 1.52i) was used for Western blot data analysis. Prism software (Version 5, GraphPad) were used for statistical analysis.

For manuscripts utilizing custom algorithms or software that are central to the research but not yet described in published literature, software must be made available to editors and reviewers. We strongly encourage code deposition in a community repository (e.g. GitHub). See the Nature Research guidelines for submitting code & software for further information.

Data

Policy information about availability of data

All manuscripts must include a data availability statement. This statement should provide the following information, where applicable:
- Accession codes, unique identifiers, or web links for publicly available datasets
- A list of figures that have associated raw data
- A description of any restrictions on data availability

The authors declare that the data supporting the findings of this study are available within the paper and its supplementary information files. Source data are provided with this paper. Data are available from the corresponding author upon reasonable request.

Field-specific reporting

Please select the one below that is the best fit for your research. If you are not sure, read the appropriate sections before making your selection.

- [x] Life sciences
- [ ] Behavioural & social sciences
- [ ] Ecological, evolutionary & environmental sciences

For a reference copy of the document with all sections, see nature.com/documents/nr-reporting-summary-flat.pdf

Life sciences study design

All studies must disclose on these points even when the disclosure is negative.

Sample size	No statistical methods were used to predetermine sample sizes. Sample size were predetermined on the basis of published studies (Wu et al, Cell, 2012. PMID: 22726441; Li et al, J Neurosci, 2012. PMID: 22972992). All quantitative experiments in this study were repeated at least three times. For in vivo experiments, the number of embryos subjected to a particular experiment are defined by the number of embryos available in the mice operated and the statistical requirements.
Data exclusions	Data were only excluded for failed experiments.
Replication	All experiments in this study were replicated at least three times with the same experimental protocol, followed by the same analysis. All replications were successful and showed the same results.
Randomization	All animals and wells of cultured cells were assigned to groups randomly.
Blinding	Investigators were not blinded to experimental groups during this study because proper controls were used.

Reporting for specific materials, systems and methods

We require information from authors about some types of materials, experimental systems and methods used in many studies. Here, indicate whether each material, system or method listed is relevant to your study. If you are not sure if a list item applies to your research, read the appropriate section before selecting a response.

Materials & experimental systems

n/a	Involved in the study	
☐	☒	Antibodies
☐	☒	Eukaryotic cell lines
☒	☐	Palaeontology and archaeology
☐	☒	Animals and other organisms
☒	☐	Human research participants
☒	☐	Clinical data
☒	☐	Dual use research of concern

Methods

n/a	Involved in the study	
☒	☐	ChIP-seq
☒	☐	Flow cytometry
☒	☐	MRI-based neuroimaging

Antibodies

Antibodies used

Commercial primary antibodies for immunoblotting are:
α-tubulin (1:10000; Sigma T5168, lot 038M4813V), α-TubK40ac (1:50000; Sigma T7451, lot 051M4770), H3K36me3 (1:10000; Abcam ab9050, lot GR3198867-1), SETD2 (1:500; Sigma HPA042451, lot F106641), Tuj1 (1:1000; Abcam ab107216, lot GR304197-2), Flag (1:1000; Sigma F7425/F3165, lot 030M4800/069K6006), PH3 (1:1000; Millipore 06-570), β-actin (1:1000; Chemicon MAB1501, lot 3282532), GAPDH (1:10000; Abcam ab8245, lot GR3275542-3), and GST (1:1000; Abcam ab6612, lot GR32259-1).
Commercial secondary antibodies for immunoblotting are:
Goat anti-mouse IgG HRP conjugated (Chemicon AP308P) and Goat anti-rabbit IgG HRP conjugated (Chemicon AP307P). HRP-coupled secondary antibodies were used at a dilution of 1:5000.
Commercial primary antibodies for immunostaining are:
α-tubulin (1:10000; Sigma T5168, lot 038M4813V), β-tubulin (1:10000; Sigma T5293, lot 043M4765), SETD2 (1:1000; Sigma HPA042451, lot F106641), GFP (1:2000; Abcam ab13970, lot GR3190550-19), Tuj1 (1:1000; Abcam ab107216, lot GR304197-2), Ki67 (1:500; BD Biosciences 550609, lot 8302515), PH3 (1:1000; Millipore 06-570), cleaved caspase 3 (1:1000; CST 9661S, lot 0037), SMI-312 (1:1000; Biolegend 837904, lot B279610), Flag (1:1000; Origene TA50011-100, lot W031), Nestin (1:1000; Millipore MAB353, lot 1970329) and Tyr-α-tubulin (1:1000; Abcam ab6160, lot GR146886-3).
Commercial secondary antibodies for immunstaining are:
Alexa Fluor 488 anti-Mouse (Thermo Fisher A32766, Donkey), Alexa Fluor 488 anti-Rabbit (Thermo Fisher A32790, Donkey), Alexa Fluor 488 anti-Chicken (Thermo Fisher A32931, Goat), Alexa Fluor 555 anti-Mouse (Thermo Fisher A32773, Donkey) and Alexa Fluor 555 anti-Rabbit (Thermo Fisher A32794, Donkey). Fluorescent labelled secondary antibodies were used at a dilution of 1:1000.
Homemade antibody: α-TubK40me3 (1:1000 for immunostaining, rabbit).

Validation	All commercial antibodies were validated for use in immunoprecipitation, immunoblotting or immunostaining by the manufacturers including Thermo Fisher, Millipore, Sigma, Abcam, BD Biosciences, CST, Chemicon, Biolegend and Origene. Full validation statements are available on manufacturers' websites<br>Homemade antibody was validated for immunoprecipitation, immunostaining, Dot blot and RNAi in Extended Data Figure 1 and 2.

Eukaryotic cell lines

Policy information about cell lines

Cell line source(s)	HEK293 cell: GNHu 18, Shanghai cellbank, Chinese Academy of Sciences.<br>Neuro-2a cells: SCSP-5035, Shanghai cellbank, Chinese Academy of Sciences.<br>ND7/23 cells: SCSP-5026, Shanghai cellbank, Chinese Academy of Sciences.
Authentication	The authors did not authenticate the referred cell lines.
Mycoplasma contamination	The authors did not test mycoplasma contamination.
Commonly misidentified lines<br>(See ICLAC register)	No commonly misidentified cell line was used in this study.

Animals and other organisms

Policy information about studies involving animals; ARRIVE guidelines recommended for reporting animal research

Laboratory animals	The following mouse strains including wild type C57BL/6, Setd2 Flox and MEC-17 knockout were used. Both male and female mice were analyzed at embryonic and postnatal stages. All mice were maintained under a 12 hours dark/light cycle, at an ambient temperature of around 22 degrees and humidity of 50%.
Wild animals	The study did not involve wild animals.
Field-collected samples	The study did not involve samples collected from the field.
Ethics oversight	All animal experiments were approved by the Institutional Animal Care and Use Committee, CAS Center for Excellence in Molecular Cell Science, Chinese Academy of Sciences.

Note that full information on the approval of the study protocol must also be provided in the manuscript.
